# Assessing the Likelihood of Transmission of *Candidatus* Liberibacter solanacearum to Carrot by Potato Psyllid, *Bactericera cockerelli* (Hemiptera: Triozidae)

**DOI:** 10.1371/journal.pone.0161016

**Published:** 2016-08-15

**Authors:** Joseph E. Munyaneza, Tariq Mustafa, Tonja W. Fisher, Venkatesan G. Sengoda, David R. Horton

**Affiliations:** USDA-ARS, Yakima Agricultural Research Laboratory, Wapato, WA, 98951, United States of America; University of Idaho, UNITED STATES

## Abstract

‘*Candidatus* Liberibacter solanacearum’ (Lso) is a phloem-limited bacterium that severely affects important Solanaceae and Apiaceae crops, including potato, tomato, pepper, tobacco, carrot and celery. This bacterium is transmitted to solanaceous species by potato psyllid, *Bactericera cockerelli*, and to Apiaceae by carrot psyllids, including *Trioza apicalis* and *Bactericera trigonica*. Five haplotypes of Lso have so far been described, two are associated with solanaceous species and potato psyllids, whereas the other three are associated with carrot and celery crops and carrot psyllids. Little is known about cross-transmission of Lso to carrot by potato psyllids or to potato by carrot psyllids. Thus, the present study assessed whether potato psyllid can transmit Lso to carrot and whether Lso haplotypes infecting solanaceous species can also infect carrot and lead to disease symptom development. In addition, the stylet probing behavior of potato psyllid on carrot was assessed using electropenetrography (EPG) technology to further elucidate potential Lso transmission to Apiaceae by this potato insect pest. Results showed that, while potato psyllids survived on carrot for several weeks when confined on the plants under controlled laboratory and field conditions, the insects generally failed to infect carrot plants with Lso. Only three of the 200 carrot plants assayed became infected with Lso and developed characteristic disease symptoms. Lso infection in the symptomatic carrot plants was confirmed by polymerase chain reaction assay and Lso in the carrots was determined to be of the haplotype B, which is associated with solanaceous species. EPG results further revealed that potato psyllids readily feed on carrot xylem but rarely probe into the phloem tissue, explaining why little to no Lso infection occurred during the controlled laboratory and field cage transmission trials. Results of our laboratory and field transmission studies, combined with our EPG results, suggest that the risk of Lso infection and spread between psyllid-infested solanaceous and Apiaceae crops is likely to be negligible under normal field conditions.

## Introduction

The potato/tomato psyllid, *Bactericera cockerelli* (Šulc) (Hemiptera: Triozidae), is a well known vector of the fastidious bacterium ‘*Candidatus* Liberibacter solanacearum’ (Lso), the putative causal agent of zebra chip disease of potato (*Solanum tuberosum* L.). Zebra chip has caused millions of dollars in losses to the potato industry in the United States, Mexico, Central America, and New Zealand [1–3]. This phloem-limited plant pathogen also severely damages other economically important solanaceous crops, including tomato (*Solanum lycopersicum* L.), pepper (*Capsicum annuum* L.), eggplant (*Solanum melongena* L.), tobacco (*Nicotiana tabacum* L.), and tamarillo (*Solanum betaceum* L.) [2–3]. The potato psyllid is believed to be a native of southwestern United States and northern Mexico, and its current geographic distribution in the New World is limited to western United States and Canada, Mexico, and Central America [4–5]. *Bactericera cockerelli* was accidentally introduced in New Zealand, where it was first documented in 2006 [6–7]. Zebra chip was shown to be associated with the potato psyllid in 2006 by Munyaneza et al. [1], preceding the discovery of Lso in 2008 [8–9].

Following the discovery of Lso economically affecting potato and other solanaceous crops in the Americas and New Zealand, Lso was documented for the first time in Europe in carrot (*Daucus carota* L.) crops and in the carrot psyllid *Trioza apicalis (= Dyspersa apicalis)* Förster, from plant and insect samples collected in Finland [10–11]. The bacterium was then reported in carrot and *T*. *apicalis* in several other European countries, including Norway, Sweden, and Germany [12–14]. Subsequently, Lso was detected in carrot and celery (*Apium graveolens* L.) crops and in the psyllid *Bactericera trigonica* Hodkinson in several countries within the Mediterranean region, including Spain, the Canary Islands, and France [15–20]. Most recently, Lso was reported for the first time on the African continent, in carrot crops in Morocco [21]. Damage to carrots by Lso-infected psyllids in Europe and northern Africa can cause up to 100% crop loss [2, 10–11, 20, 22]. Furthermore, Lso was recently shown to be transmitted through carrot seeds [23].

While several psyllid species in Europe are known to have carrot as a host plant, no psyllids in the Americas or New Zealand have been documented to have this plant species as a host. Potato psyllid, a known host of Lso, is not present in Europe [24–25]. In addition, five geographic haplotypes of Lso have so far been described, including two (A and B) that are associated with solanaceous species and potato psyllids [26] and three (C, D, and E) that are associated with carrot and celery crops and carrot psyllids [20, 27]. The five Lso haplotypes are not yet known to elicit biological differences in the plant or insect hosts and their apparent stability suggests separate long-lasting populations of the bacterium [26, 28]. Therefore, it is not known whether Lso haplotypes associated with solanaceous species can successfully infect and induce disease symptoms in Apiaceae and vice-versa.

Several studies have demonstrated transmission of Lso by *B*. *cockerelli* to potato and other solanaceous species [8, 29–35] and by *T*. *apicalis* and *B*. *trigonica* to carrot [22, 20]. However, little is known about cross-transmission of Lso to carrot by potato psyllid or to potato by carrot psyllids, or whether cross-transmission could actually lead to successful development of symptoms characteristic of the bacterium infection in either crop. This is particularly important, given the potential risk of Lso infection and spread between solanaceous and Apiaceae crops that might be growing in close proximity. Thus, the objectives of the present study were to: 1) assess whether potato psyllid can transmit Lso to carrot under controlled laboratory and field conditions, 2) assess whether Lso haplotypes infecting solanaceous species can also infect carrot and induce characteristic disease symptoms, and 3) assess stylet probing behavior of potato psyllid on carrot with electropenetrography (EPG) technology to further elucidate potential Lso transmission to Apiaceae by this potato insect pest.

## Materials and Methods

### Sources of Insects

Lso-free and Lso-infected potato psyllid colonies were established at the USDA-ARS facility in Wapato, WA (46° 26′ 44″ N, 120° 25′ 19″ W), with insects collected from a potato field in Dalhart, Texas (36° 3′ 44″ N, 102° 31′ 24″ W) in 2007. No insect collection permit was required; potato psyllid is not an endangered or protected species. The insects were maintained on Atlantic potatoes under laboratory conditions of 25±1°C, 40±5% RH, and a photoperiod of 16:8 (L: D) h. The colonies were later confirmed to be composed of psyllids of the Central haplotype, using high resolution melting analysis [36]. The psyllid colonies were tested for Lso on a monthly basis by polymerase chain reaction (PCR), and the infection rate in the Lso-infected colonies ranged between 80 and 100% at the time of the experiments.

### Sources of Plants

Certified disease-free potato mini-tubers (var. Atlantic) were obtained from CSS Farms Inc. (Colorado City, CO); this variety was selected due to its high susceptibility to zebra chip [37]. Similarly, certified disease-free seeds of carrot (var. Danvers Half Long) were obtained from Ed Hume Seeds, Inc. (Puyallup, WA). Potato and carrot plants were grown in a greenhouse at the USDA-ARS Wapato facility in ½-L pots (Kord Products, Toronto, Ontario, Canada). The plant growth media consisted of a mixture of 86% sand, 13.4% peat moss, 0.5% Apex time release fertilizer (J. R. Simplot Co., Lathrop, CA), and 0.1% Micromax micronutrients (Scotts Co., Marysville, OH).

### Assessing Lso Transmission to Carrot by Potato Psyllid under Laboratory Conditions

The experiment was conducted at USDA-ARS Wapato facility in January of 2010, under greenhouse conditions. Potato plants were in growth stage III (tuber initiation) when used in the experiment, and their height ranged from 15 to 25 cm; at this growth stage, zebra chip disease symptoms are easily observed in tubers obtained from infected plants. Carrots were grown in ½ L pots from seed. Three to five carrot seeds were planted per pot. Following germination, seedlings were thinned to one plant per pot. Seedlings were at 4-leaf stage when used in the transmission experiment. All plants of both species were tested for Lso by PCR to confirm absence or presence of this pathogen before and after being used in the transmission experiments, respectively.

Carrot and potato plants were inoculated with Lso by infesting them with Lso-infected potato psyllids in a greenhouse maintained at 24–28°C, 50±5% RH, and a 16:8 (L:D) h photoperiod. The potato plants were included in the experiment to serve as positive controls. The carrot and potato plants were individually exposed to potato psyllid adults collected from the Lso-infected colony described above. Each plant was enclosed in a small hoop cage made of 40-cm metallic flag wires (Gempler’s Inc., Madison, WI) inserted into the four corners of the pot to create a frame over the plant as described by Buchman et al. [30] and Munyaneza et al. [38]. The frame was covered with an Econet SF insect screen fabric (USGR, Inc., Seattle, WA) and the net was secured to the base of the pot with a cloth-covered telephone cord (Oldphonesworks Inc., Kingston, ON, Canada) [30, 38]. Twenty adult potato psyllids were released into each cage and allowed to freely settle, feed, and reproduce on the plant up to the 4-month duration of the greenhouse experiment.

Eighty and 40 carrot and potato plants, respectively, were exposed to psyllids. An additional 15 carrot and potato plants each that had not been exposed to psyllids were included in the experiment to serve as negative controls. Following insect exposure, plants were maintained in the greenhouse at 24–28°C, 50±5% RH, and a 16:8 (L:D) h photoperiod, as described above. The plants were visually inspected for psyllid survival on a weekly basis for six weeks by first placing individual plants in a dome cage (BioQuip Products, Rancho Dominguez, CA), uncovering them, and then estimating the number of live psyllids per plant. In addition, the potato plants were monitored for development of zebra chip symptoms, which include upward rolling of the top leaves developing into a basal cupping of the leaflets, accompanied with reddish discoloration, shortened internodes, and leaf scorching, collapsed stolons, brown discoloration of the vascular ring in the tubers, necrotic flecking of internal tuber tissues, and streaking of the medullary ray tissues [1, 2]. Similarly, the carrot plants were also monitored for psyllid survival and Lso infection symptoms, including leaf curling, yellowish, bronze and purplish discoloration of leaves, stunting of the carrot shoots and roots, and proliferation of secondary roots [10, 11]. To confirm Lso infection, the potato plants were tested for the bacterium by PCR (methods described below) four to six weeks following insect exposure and at the end of the 4-month experiment for carrots; potato plants usually do not survive beyond eight weeks following Lso infection.

### Assessing Lso Transmission to Carrot by Potato Psyllid under Field Cage Conditions

In addition to the greenhouse transmission experiment, a field cage trial was conducted at the USDA-ARS Wapato facility during the summer of 2010. Carrot and potato plants were grown in small field cages and exposed to potato psyllids from an Lso-infected colony. The cages were used as enclosures to prevent escape of the potato psyllids released onto the plants and to exclude other potato pests and they were designed as described in detail previously by Buchman et al. [30]. The soil at the site is sandy loam and was treated with pre-plant herbicides S-ethyl dipropylthicarbamate (Eptam; Gowan Co., Yuma, AZ) and trifluralin (Treflan; Dow Agrosciences, LLC Calgary, AB) at label rates prior to planting for weed control. Additional weeding was done by hand as needed throughout the season. The individual plot under each cage measured 2 m long by 1 m wide and the ground was hilled into a mound ~ 0.3 m high. The frame of each cage was formed by inserting both ends of 3 m long fiberglass poles (Geotek, Inc.; Stewartville, MN) into the ground at opposite ends of the plot, to form hoops over the mounds. A third fiberglass pole was placed halfway between the outside poles forming a central support. Insect proof fabric, Econet SF (USGR, Inc.; Seattle, WA) 3 m wide and 5 m long, was draped over the frame and the edges were buried in the ground [30]. The cages were arranged in four rows of six cages each, for a total of 24 cages. Each cage was spaced approx. 1.5 m from the next one endwise and a spacing of 1 m was placed between rows.

Certified disease-free potato (var. Atlantic) and carrot (var. Danvers Half Long) seed were used to grow plants for the field trial. Eight whole potato seed tubers were planted per cage in mid-May 2010, with a spacing of 23 cm between seed tubers and planted at a depth of 13 cm. Carrot was also directly seeded in the cage mounds and, prior to insect exposure, the seedlings were thinned to 10 per cage. Fertilizer (Simplot Grower Solutions 16-16-16; Simplot Grower Solutions, Halsey, OR) was side-dressed at the time of planting at a rate of 37.4 g/cage. Drip tape irrigation (Queen Gil, Intl. Burgas, Bulgaria) was used throughout the season to water the potato and carrot plants. Eight and 16 cages were planted with potato and carrot, respectively.

Twenty psyllids from the Lso-infected colony were released onto each potato plant in the cages in mid-June 2010, at the potato tuber initiation stage as in the laboratory trial. This plant stage was again selected to ensure tuber production and visual assessment of characteristic zebra chip symptoms in tubers. Plants in four cages were exposed to psyllids, whereas the remaining four cages remained psyllid-free and served as controls. As for carrot, similarly to the laboratory trial, the plants were also exposed to psyllids at the 4-leaf stage in mid-June 2010. Because we had noticed during the laboratory trial that potato psyllid does not reproduce on carrot, each carrot plant was first exposed to 50 psyllids, followed by an additional release of 50 psyllids per plant three weeks later; this was done to increase the insect pressure and the chance of Lso inoculation. Psyllid release on carrot was made in 12 cages, with the remaining four cages kept psyllid-free to serve as controls. Monitoring of psyllid numbers in the field cages was not practical, so survival rates were not estimated. As in the laboratory transmission trial, the carrot and potato plants were monitored for characteristic symptoms of Lso infection and tested for the bacterium by PCR at the end of the experiment, to confirm pathogen infection.

### Nucleic Acids Extractions

Total DNA was extracted from carrot and potato plant material using a modified version of the cetyltrimethylammonium bromide (CTAB) extraction method of Pastrik and Maiss [39] as described in detail previously [35]. Briefly, 500 mg of carrot tissue (petioles or roots) or potato tissue (petioles, stolons, and/or tubers) was macerated in 1 mL of extraction buffer (100 mM Tris-HCl, pH 8.0, 50 mM EDTA, 500 mM NaCl, 10 mM mercaptoethanol) using BioReba sample bags and a `Homex' apparatus (Bioreba, Switzerland). Carrot or potato tissue macerate (300 μl) was mixed with 80 μl lysozyme (50 mg/ml in 10mM Tris-HCl, pH 8.0; Sigma-Aldrich, Inc., St Louis, MO) and incubated for 30 min at 37°C. After incubation, 500 μl CTAB buffer (2% CTAB, 1.4 M NaCl, 20 mM EDTA, 100 mM Tris–HCl, pH 8.0, 0.2% mercaptoethanol) was added to the homogenate and the sample was incubated for 30 min at 65°C. The sample was then placed at room temperature for 3 min before the addition of 500 μl of ice cold chloroform. Samples were vortexed to mix, then centrifuged at 21,000 x g for 10 min. The aqueous layer was then transferred to a new microfuge tube and 0.6 volume of isopropanol was added and the tube was placed on ice for 20 min to precipitate DNA. DNA was recovered by centrifugation as above. The pellet was washed with ice cold 75% ethanol and centrifuged 21,000 x g for 2 min. After removal of ethanol, the pellet was air dried then re-suspended in 100 μl sterile water.

Total DNA was also extracted from potato psyllids using the CTAB buffer extraction method of Zhang et al. [40]. Individual insects were ground in 600 μl of fresh buffer (2% CTAB, 1.4 M NaCl, 20 mM EDTA, 100 mM Tris–HCl, pH 8.0, 0.2% mercaptoethanol) using a micropestle and processed as described by Zhang et al. [40]. Nucleic acids were re-suspended in 100 μl of sterile water.

### Polymerase Chain Reaction Assay

Insect and plant DNA sample extracts were tested for the presence of Lso by conventional PCR, using primer pair OA2/OI2c (5’-GCGCTTATTTTTAATAGGAGCGGCA-3’/ 5’-GCCTCGCGACTTCGCAACCCAT-3’) [9, 41–42] which amplifies DNA sequences from the 16S rRNA gene of Lso. This primer pair is expected to produce 1168 bp-amplicon [9, 10–11, 41–42]. Negative and positive controls were included in all PCR assays. Amplifications were performed in 25 μl reactions with Green Go Taq Polymerase (Promega, Inc., Madison, WI) according to the manufacturer’s instructions. For each reaction, 10 pmol of each primer, and 1 μl of DNA extract was added, and incubated with the following conditions: initial denaturation for 3 min at 94°C, then amplification for 20 s at 94°C, 20 s at 65°C, 1 min at 72°C for 39 cycles, followed by a final 5 min at 72°C incubation. PCR products were separated on 1.5% agarose gels containing ethidium bromide for visualization.

### DNA Cloning and Sequencing Procedures

Cloning and sequencing were performed for carrot only, to conclusively confirm Lso infection. Briefly, two carrot amplicons (from two symptomatic carrot plants collected in August and October 2010, respectively; see Results section) were selected and excised using clean razor blades and ethidium bromide was removed using GenElute Minus EtBr Spin columns (Sigma-Aldrich, Inc., St Louis. MO). The purified PCR products were cloned using the TOPO TA cloning kit® (Invitrogen, Carlsbad, CA) with TOP 10 *Escherichia coli* chemically competent cells. Plasmid DNA was extracted from selected colonies using the QIAprep spin mini prep kit (Qiagen, Valencia, CA) and the DNA clones were sequenced at MC Laboratories (MCLab, San Francisco, CA).

### Electropenetrography Recordings

As a follow-up to the Lso transmission trials, the stylet probing behavior of potato psyllid on carrot was assessed using electropenetrography (EPG) technology, to further elucidate potential Lso transmission to carrot by potato psyllid. This technology has been proven to be a very useful tool to assess acquisition and inoculation of psyllid-transmitted plant pathogens, including the phloem-limited Lso [34–35, 43]. The stylet probing behavior activities by potato psyllid on carrot were compared with those of this insect vector on potato [35, 43]. EPG system wiring and recordings were conducted as described in detail previously by Pearson et al. [43] and Mustafa et al. [35]. Briefly, the potato psyllids used for EPG recordings were collected from an Lso-free colony. The insects were placed on ice for about 30 min to reduce their mobility for preparation for wiring. The immobilized psyllids were then secured by gently gripping their fore- and hind wings with a pair of soft grip forceps (BioQuip Products, Rancho Dominguez, CA). An electrode consisting of a 3-cm long copper wire soldered to the head of a 3 mm diameter brass nail was used to wire the insects to the EPG system. One end of a 3-cm long and 25.4-μm diameter gold wire (sold as a 0.0010 in.; Sigmund Cohn., Mt. Vernon, NY, USA) was attached to the psyllid pronotum using a hand-mixed silver glue adhesive that consisted of 1:1:1 (v: v: w) of water-based white household glue, water and silver flake (Inframat Advanced Materials LLC, Manchester, CT, USA). The other end of the gold wire was attached to the copper wire portion of the electrode. The wired insects were left hanging at room temperature for a 30-min recovery and wiring adjustment period on a small styrofoam pinning board and without plant material. After the recovery period, the electrode was inserted into the EPG amplifier and the tethered psyllid was placed on the abaxial surface of a leaf of a carrot or potato plant in a Faraday cage, and stylet probing behavior was recorded for 24 h. The EPG recordings were performed in an experimental room maintained at 25 ± 1°C, 40 ± 5% RH, with a photoperiod of 16:8 (L:D) h. EPG waveforms were acquired using a four-channel AC-DC EPG monitor (EPG Equipment Co., Otterville, MO) [44]; thus, recordings could be done simultaneousily for a maximum of four psyllids (two on carrot and potato plants each). The EPG data were digitized using a WinDaq DI-720 analog-to-digital (A-D) board and recorded with WinDaq Pro+ acquisition software (DATAQ Instruments, Akron, OH) at a sample rate of 100 Hz, input impedances (Ri) of 10^9^ Ohms (Ω), and DC substrate voltage.

Total number of waveform or probing events and time duration spent in xylem sap ingestion (G), initial contact with phloem tissue (D), salivation into phloem sieve elements (E1), and phloem sap ingestion (E2) were recorded for psyllids on both carrot and potato plants. Thirty eight psyllids were assayed, comprising 23 psyllids on carrot and 15 psyllids on potato.

### Data Analysis

Statistical analysis was conducted for the EPG data. The average duration and number of probing behavior events of xylem ingestion were compared among psyllids feeding on carrot and potato plants using GLIMMIX procedure of SAS 9.3 [45]. Data were examined for heterogeneity of variance using residual plots and for non-normality of errors using normal quantile-quantile plots. Based on plots, the DIST = LOGN option of the MODEL statement was included for analyses comparing xylem-ingestion among psyllids feeding on carrot and potato plants [46]. The proportion of psyllids that exhibited a specific probing behavior was compared between plant species using Fisher’s exact tests (Proc FREQ⁄ FISHER; [45]).

## Results

### Lso Transmission under Laboratory Conditions

All potato plants exposed to psyllids under greenhouse controlled conditions developed ZC symptoms and tested positive for Lso by PCR, four weeks following insect exposure. In contrast, none of the carrot plants exposed to psyllids exhibited Lso infection symptoms or tested positive for the bacterium, up to four months after insect exposure. As expected, none of the negative control potato or carrot plants (no psyllid exposure) exhibited Lso infection symptoms or tested positive for the bacterium. Psyllids readily survived and reproduced on potato plants until the termination of the experiment (5–6 weeks following insect exposure), as expected. Interestingly, psyllids also did survive on carrot plants and over 10 live psyllids per plant were observed for at least six weeks; however, no psyllid reproduction (eggs or nymphs) occurred on the carrot plants during the experiment 4-month duration.

### Lso Transmission under Field Cage Conditions

**Fig 1:**
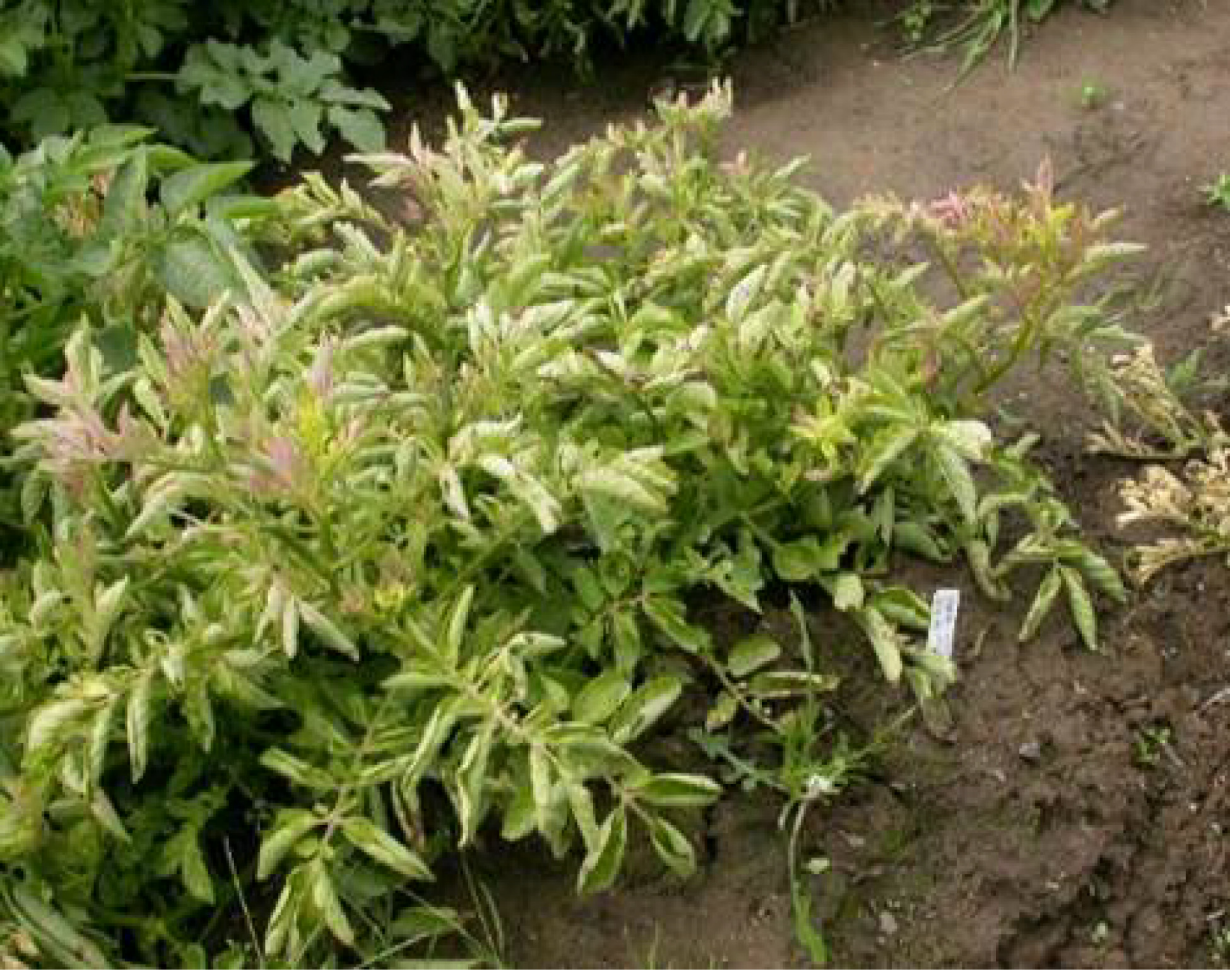
A potato plant exhibiting typical zebra chip disease foliar symptoms, following exposure to ‘*Candidatus* Liberibacter solanacearum’ (Lso)-infected potato psyllids (*Bactericera cockerelli*) under controlled field cage conditions.

**Fig 2:**
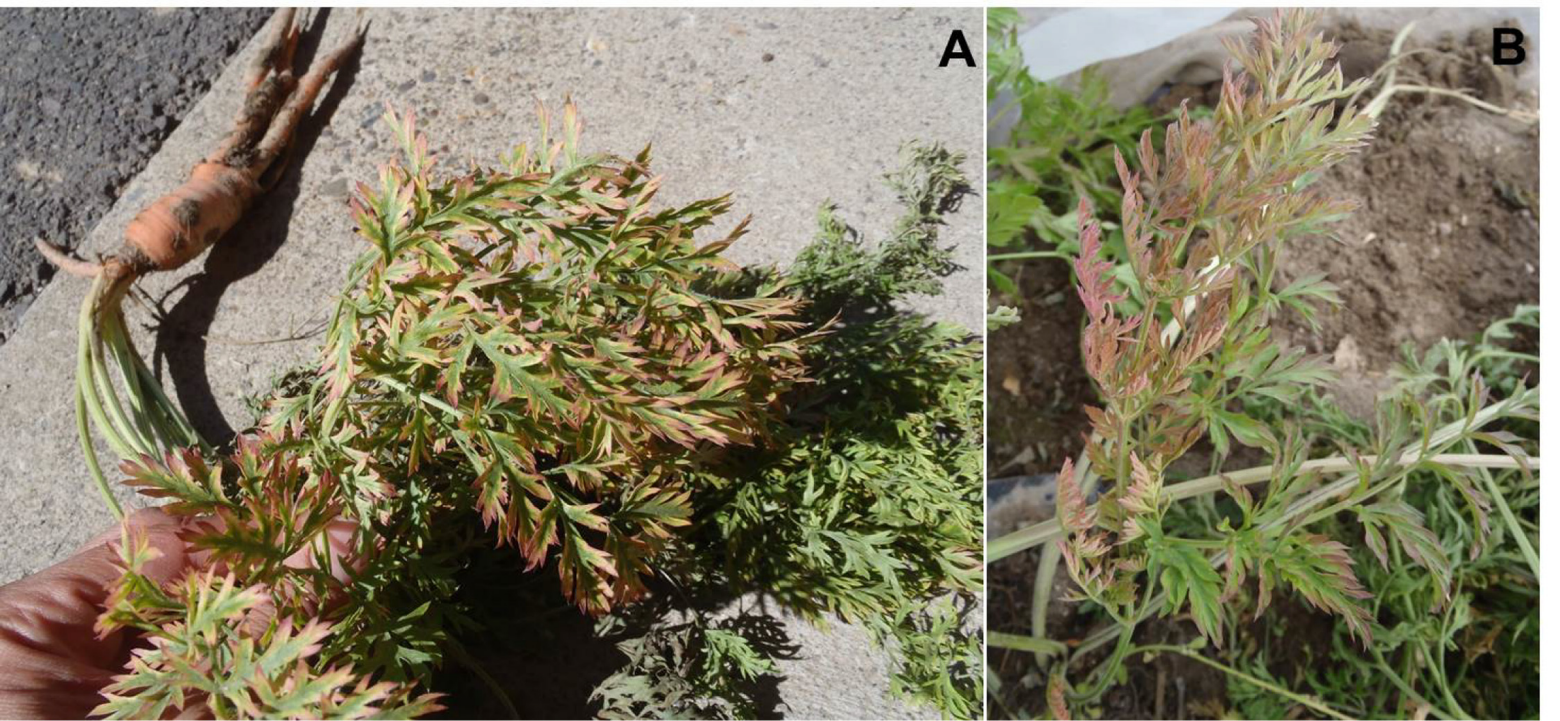
Carrot plants exhibiting characteristic symptoms of ‘*Candidatus* Liberibacter solanacearum’ (Lso) infection, following exposure to Lso-infected potato psyllids (*Bactericera cockerelli*) under controlled field cage conditions. The symptomatic plants were collected in August (A) and October (B) 2010, respectively, from a field trial conducted at USDA-ARS Wapato facility.

**Fig 3:**
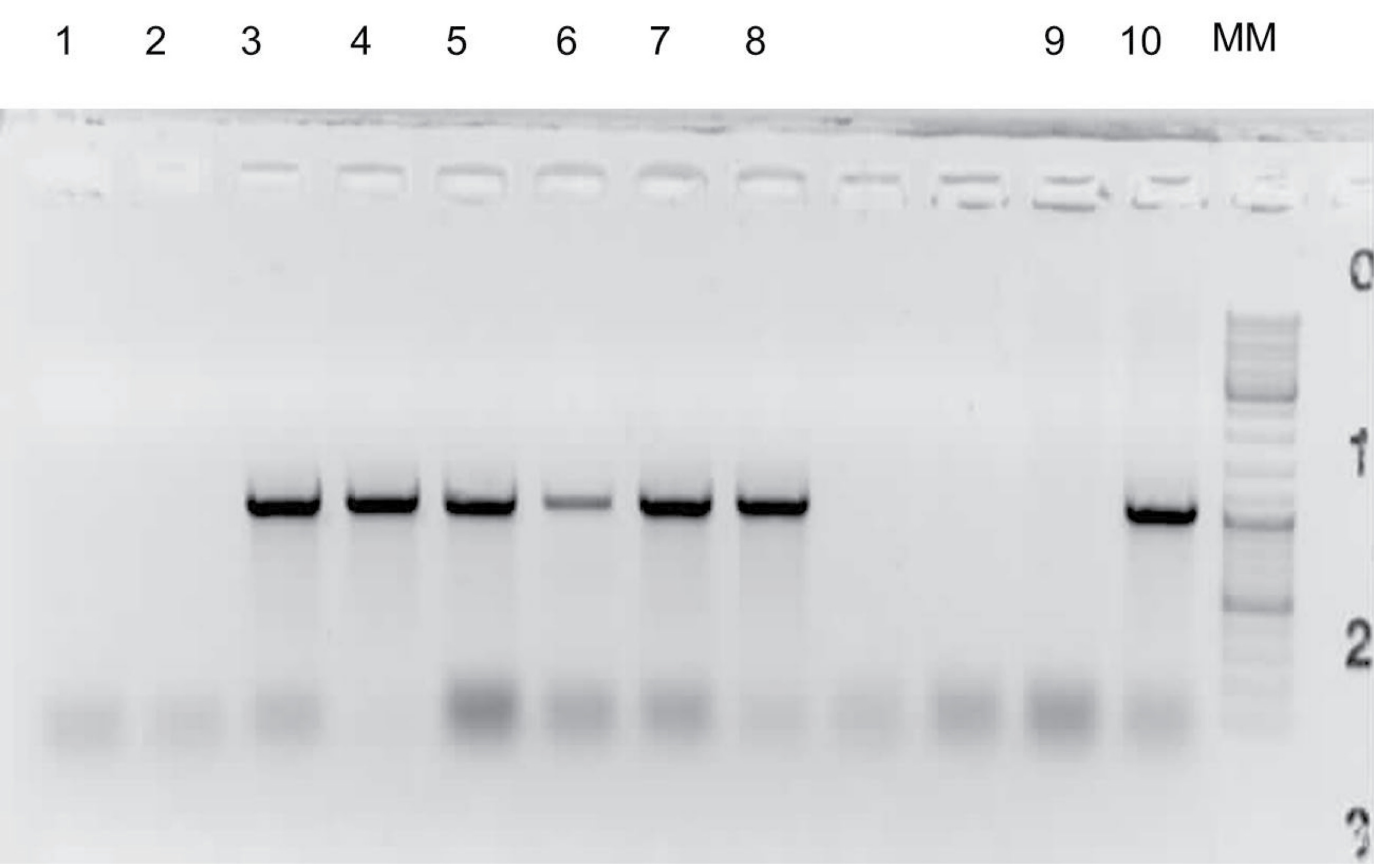
PCR detection of ‘*Candidatus* Liberibacter solanacearum’ (Lso) 16S rRNA gene in genomic DNA extracted from Lso-free potato tubers (lanes 1 and 2), symptomatic carrot root tissue (lanes 3, 4, and 5), symptomatic carrot petiole tissue (lanes 6, 7, and 8), Lso-free potato psyllid (*Bactericera cockerelli*) (lane 9), and Lso-infected potato psyllid (lane 10) by using species-specific primer pair OA2/O12c [42].

Similarly to the laboratory transmission experiment, all 32 potato plants exposed to psyllids in the field cages developed typical ZC symptoms (Fig 1), 3–4 weeks following insect exposure, and tested positive for Lso by PCR. None of the 32 potato plants in the control cages developed ZC symptoms or tested positive for Lso. Three of the 120 carrot plants exposed to psyllids in the field cages developed symptoms resembling those caused by Lso infection (Fig 2), comprising one plant on 20 August and two plants on 25 October 2010 (two and four months, respectively, following exposure to infected psyllids). None of the 40 carrot plants in the control cages exhibited symptoms of Lso infection or tested positive for the bacterium. DNA was extracted from the three symptomatic carrot plants and PCR testing revealed that the plants were indeed infected with Lso (Fig 3). PCR amplicons of two of the symptomatic plants (one from each of the two collection dates) were cloned and four clones from each of the two amplicons were sequenced. Two consensus sequences were generated by aligning the 4 sequences from the clones of each of the two carrot plant samples. The two consensus sequences were 100% identical and were submitted to the GenBank database (Accession No. KU588194 for the carrot plant collected in August and Accesion No. KU588195 for the carrot plant collected in October). BLAST analysis of the 16S rDNA carrot consensus sequences showed 100% homology with those of Lso previously amplified from potato, including GenBank Accession Nos. FJ498806, JX559780, and HM245242 from Mexico, Texas, and Washington, respectively, and EU921627 from potato psyllids collected from Dalhart, Texas (S1 Table). These carrot consensus sequences were also 99.7% identical to several 16S rDNA sequences from carrot in the GenBank database, including Accession Nos. GU373049, JN863095, and JN863097 from Finland, Sweden, and Norway, respectively, and GU477254 and GU477255 from the carrot psyllid *T*. *apicalis* from Finland (S1 Table). In addition, the two sequences had 99.6% homology with numerous sequences previously amplified from carrot and the psyllid *B*. *trigonica* from the Mediterranean region, including Accession Nos. HQ454312, HQ454316, and HQ454302 from the Canary Islands and mainland Spain (S1 Table). Furthermore, the sequences were aligned with those of Lso haplotypes A and B [26, 47] and it was determined that the Lso in the inoculated carrot plants was of haplotype B, which is associated with solanaceous plants and potato psyllid (S2 Table).

### Assessment of Stylet Probing Behavior of Potato Psyllid on Carrot and Potato Using Electropenetrography

**Fig 4:**
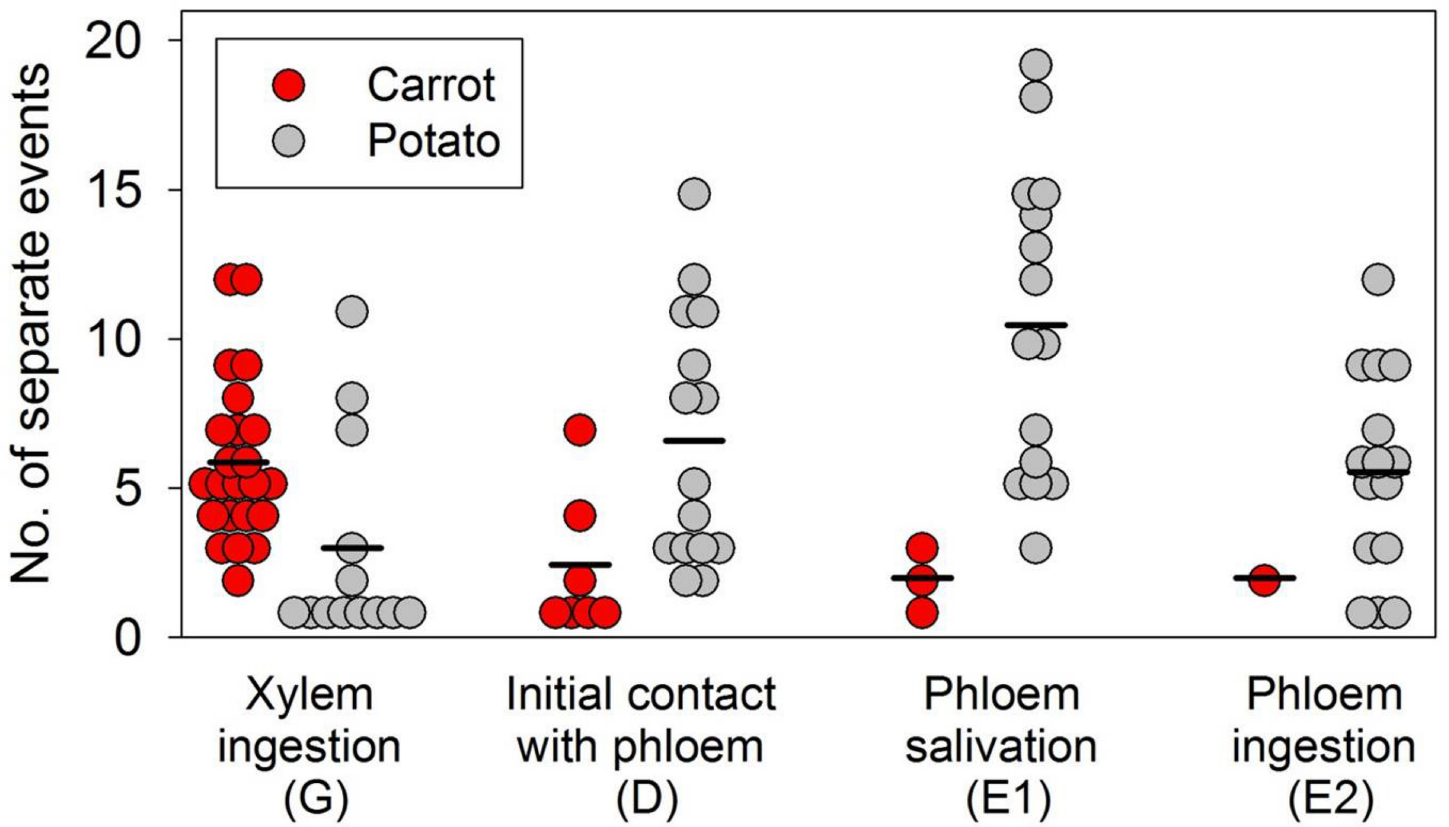
**Number of different stylet probing events per potato psyllid on carrot (red circle) or potato (gray circle) over the duration of a 24 h electropenetrography assay; the bar (-) represents the average events per each behavior and plant species.** While almost all the psyllids on potato performed all the stylet probing behaviors multiple times, only three psyllids salivated into the carrot phloem tissue, whereas only one individual ingested the carrot phloem.

Electropenetrography (EPG) showed that all psyllids in the carrot treatment reached and ingested from the xylem (G) at least once within the 24 h recording interval (23 of 23 psyllids; Fig 4, red circles). In contrast, the number of psyllids on carrots which reached phloem tissues (D) was only 30% of assayed insects (Fig 4, red circles; psyllids failing to engage in the activity were excluded from the graph). The probability of a psyllid on carrot salivating into phloem tissue (E1) and ingesting from those tissues (E2) was even lower, at 13% and 4% of insects, respectively (Fig 4, red circles). Virtually all of the 15 psyllids that were assayed on potato engaged in all four activities at least once during the 24 hour monitoring period (Fig 4, gray circles). Fisher’s exact tests showed that proportion of psyllids exhibiting contact with phloem (D), phloem salivation (E1), or phloem ingestion (E2) was significantly higher for the potato treatment than the carrot treatment (P < 0.0001 for each of the three tests).

**Fig 5:**
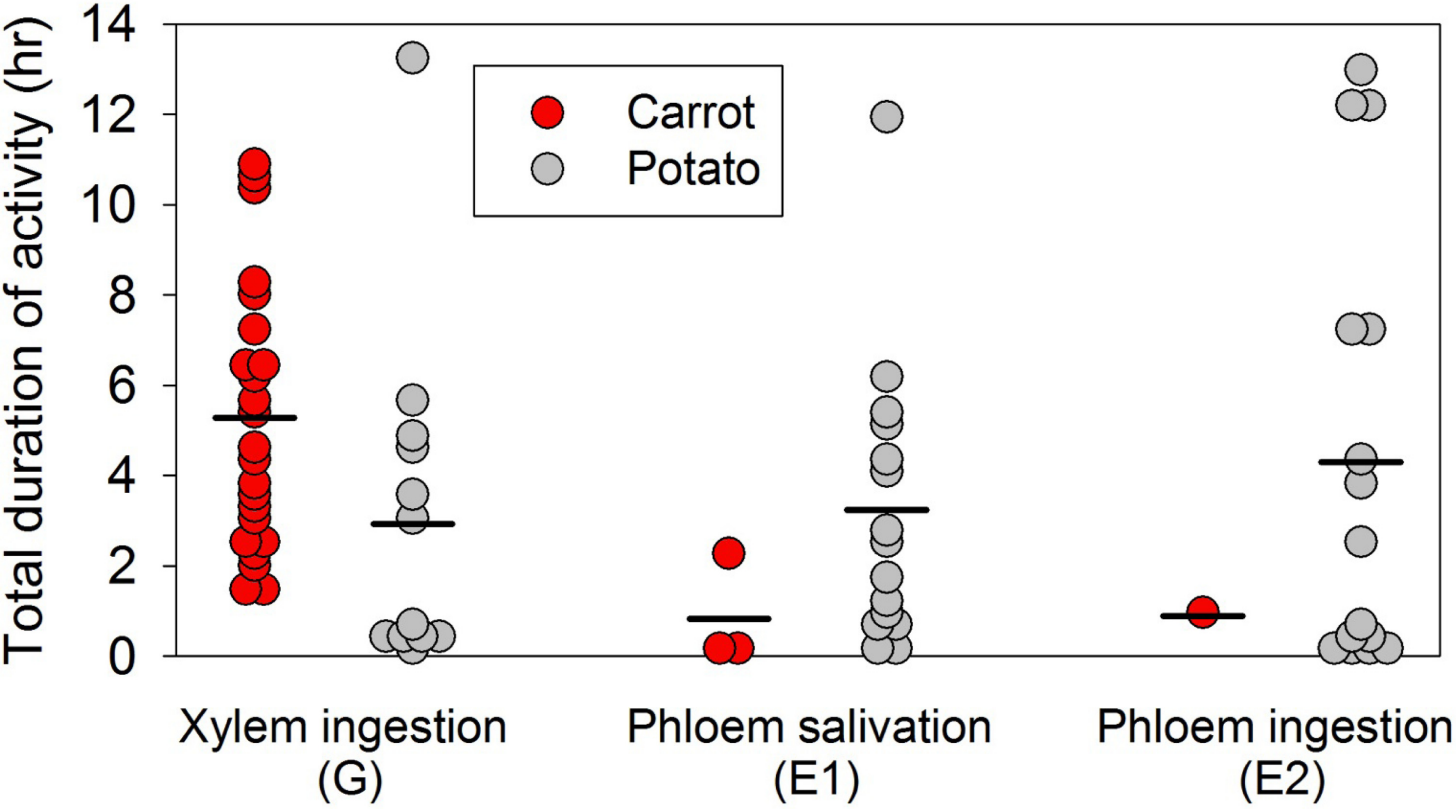
**Total time spent in the different stylet probing behavior per potato psyllid on carrot (red circle) or potato (gray circle) over the duration of a 24 h electropenetrography assay; the bar (-) represents the average duration per each behavior and plant species.** Psyllids readily ingested the xylem tissue of both carrot and potato and for long durations. While all the psyllids on spent substantial time ingesting and salivating into the potato phloem tissue, only one psyllid spent about 2 h salivating into the carrot phloem tissue, whereas another spent 1 h ingesting the phloem tissue of carrot.

All the potato psyllids readily probed and fed on xylem tissue of potato and carrot multiple times and for long durations. The psyllids on carrot ingested the xylem sap more times than those on potato, with an average of 5.9 and 2.6 probing events during the 24 h of EPG recordings, respectively (F_1, 34_ = 20.43, P = <0.0001) (Fig 4). Similarly, potato psyllids on carrots spent more time ingesting the xylem sap than those on potato, with an average duration of 5.3 and 2.5 h, respectively (F_1, 34_ = 13.17, P = 0.0009) (Fig 5). Also, there were significant differences in initial contact with phloem tissue (P< 0.0001), phloem salivation (P< 0.0001, and phloem ingestion (P< 0.0001) between psyllids on carrot and those on potato (Figs 4 and 5). While all of the 15 potato psyllids on potato made initial stylet contact with the phloem tissue, only seven out of 23 psyllids on carrot attempted to do so (Fig 4) and only three potato psyllids successfully salivated into the carrot phloem tissue (1 to 3 attempts) for very short periods of time (4–5 min), with the exception of a single individual that spent 2.3 h into this stylet probing behavior activity (Figs 4 and 5). In contrast, almost all the psyllids on potato salivated into the phloem multiple times, with an average of 10.5 salivation events per insect (Fig 4). These psyllids also spent substantial time salivating into the phloem tissue, with an average of 3.2 h (Fig 5). Similarly, while all the psyllids on potato ingested the phloem sap, with an average of 5.5 probing events per insect during the 24 h EPG recording period, only a single potato psyllid ingested the carrot phloem sap twice for a total duration of about 1 h (Figs 4 and 5). In contrast, most of the psyllids on potato spent substantial time ingesting the phloem sap, with an average of 4.3 h (Fig 5).

## Discussion

‘*Candidatus* Liberibacter solanacearum’ is an economically important bacterial pathogen of Solanaceae and Apiaceae affecting potato, tomato, pepper, tobacco, carrot and celery crops in the Americas, Europe, Africa, and New Zealand. This phloem-limited bacterium is transmitted to potato and other solanaceous species by potato psyllid, *B*. *cockerelli*, and to carrot and celery crops by the carrot psyllids *T*. *apicalis* and *B*. *trigonica*. There is no geographic overlap between *B*. *cockerelli* and the carrot psyllids. Potato psyllid is currently restricted to North and Central America, and to New Zealand. The two carrot psyllids are widespread in Europe and northern Africa. No psyllids having Apiaceae as hosts have been reported from the Americas or New Zealand (24, 48]. Differences between crops in psyllid complexes may extend to differences in Lso complexes. Five geographic/host plant haplotypes of Lso have been described, comprising two haplotypes (A and B) associated with solanaceous plant species, and three haplotypes (C, D, and E) associated with carrot and celery crops [20, 26–28]. Haplotype A has been found in solanaceous crops from Central America through western Mexico and the western United States, and in New Zealand, whereas haplotype B is currently known from eastern Mexico northwards into the central United States [26]. Haplotype C has been found in carrots in Finland, Sweden, Norway, and Germany [14, 26–28], while haplotypes D and E were recently described from infected carrot and celery crops and the psyllid *B*. *trigonica* in Spain, the Canary Islands, and Morocco [20–21, 28].

Concerns have been raised that fields of carrots growing in the vicinity of psyllid-infested potato or other solanaceous crops in the Americas and New Zealand could become infected with Lso if infected potato psyllids were to disperse onto the carrot crops from the potato fields (Munyaneza, unpublished data). We used two approaches to examine whether potato psyllid might be a threat to carrot. First, we conducted cage trials in which carrot was exposed to infective potato psyllids under no-choice conditions. Our initial trial, conducted in a laboratory setting and consisting of 80 carrot plants (20 psyllids per plant), failed to produce infection. We next conducted a larger trial under field conditions. Plants were exposed to unrealistically high densities of infected psyllids (at least 50 insects per plant) for an extended period of time (over six weeks). Even with this pressure, only three of 120 plants assayed developed characteristic disease symptoms (Fig 2). Lso infection in those three plants was confirmed by PCR and sequencing of DNA. The bacterium in the symptomatic carrot plants was found to be of the haplotype B. This is the first evidence that an Lso haplotype (i.e., haplotype B) currently assumed to be associated only with solanaceous species and potato psyllid is able to establish and induce disease symptoms in a non-solanaceous plant species.

Second, we used electropenetrography studies to compare feeding behavior of potato psyllid on carrot to those same behaviors by psyllids on potato. Electropenetrography assays have shown that acquisition of Lso by potato psyllid and subsequent inoculation into potato plants occur during the phloem sap ingestion and salivation phases, respectively, of the psyllid’s stylet probing activities [34–35, 43]. Thus, we examined whether the apparent difficulties in inoculating Lso into carrot by potato psyllid shown in the cage studies might be explained by the probing activities of the psyllid. Our comparison of probing behavior on carrot versus potato revealed that potato psyllids readily fed on carrot xylem, to the extent that psyllids on carrot spent more time in xylem tissues than psyllids on potato. This observation may help explain the somewhat surprising longevity of potato psyllid adults in the cage studies when confined to carrot. Thus, while the psyllid failed to reproduce on carrot, indicating that carrot is not a suitable reproductive host, the adult psyllid did survive for extended periods of time on carrot (up to six weeks). We assume that resources associated with the xylem tissues of carrot, and presumably ingested by the psyllid, contributed to longevity of the psyllid on carrot.

Of more interest in understanding the potential threat to carrot by potato psyllids is information about whether potato psyllids actually feed in phloem tissues of carrot. While several (7 of 23) psyllids on carrot did make stylet tip contacts with phloem tissues during their probing activities (Fig 4D), most (20 of 23) of these psyllids pulled stylets out of the tissue apparently without salivation or ingestion. Two of the psyllids that salivated into phloem did so for a duration of less than 5 min; only a single psyllid salivated into the phloem for more than 5 min (about 2 h; Fig 5E1). Only one psyllid actually ingested carrot phloem sap, spending approximately 1 h in this behavior (Fig 5E2). In contrast, all of the psyllids on potato spent substantial time salivating into and ingesting from phloem tissues (Fig 5E1 and 5E2, gray symbols). These observations indicate that most of the potato psyllids avoided feeding in phloem tissues of carrot, despite having no difficulties feeding in xylem tissues. Mustafa et al. [35] demonstrated that it may take a duration as short as 5 min for potato psyllid to inoculate Lso into potato plants once the insect stylets have reached the phloem tissue, leading to development of zebra chip symptoms in potato. While most of the psyllids on carrot failed to probe into the phloem tissue, one psyllid of the 23 that were assayed did spend over 2 h salivating into the carrot phloem tissue. The rare occurrence of this behavior by psyllids confined to carrot may explain the very occasional instance of transmission of Lso to carrot that we observed in the field cage study (3 of 120 plants assayed).

For several reasons, we suggest that potato psyllid is unlikely to be a source of Lso in carrot crops under normal field conditions. First, it is unlikely that potato psyllid would regularly colonize and settle on carrot, a non-host species, under normal field conditions. In the absence of host plants, carrot conceivably could act as a temporary food plant [48–51], as indicated by the psyllid’s willingness to feed extensively in carrot xylem (Figs 4 and 5) and by its ability to survive for several weeks on carrot in controlled no-choice cage conditions. However, given the opportunity to disperse (unlike what was allowed by our cage studies), potato psyllids that might visit a carrot crop in a field situation are unlikely to exhibit long-duration residency on that plant species. Second, our cage studies were designed to maximize opportunity of transmission rather than to mimic actual infection pressure. Incidence of Lso in potato psyllids under field conditions is generally very low (about 3%, [52]), thus psyllids that might actually colonize carrot under field conditions are very likely to be free of Lso. The very low rate of infection in field populations of psyllids is in stark contrast to the very high infection rate (80–100%) of psyllids used in our cage tests. Third, even should infective potato psyllid colonize carrots in the field, our EPG results indicate that probability that the infective psyllids would actually salivate into phloem (a necessary behavior for transmission of Lso) is very low. The EPG trials showed that potato psyllids only very rarely fed on the phloem tissue of carrot in no-choice assays. Thus, probability that this phloem-limited bacterium would be transmitted to carrot following colonization by an infected psyllid would appear also to be very low.

In sum, results from our cage studies and EPG assays, combined with our understanding of the biology of this psyllid, leads us to conclude that potato psyllid is unlikely to transmit Lso to carrots under normal field conditions. Whether Lso might spread in the opposite direction, i.e, from infected carrot crops to potato, has not been determined. However, the methods used here to examine transmission of Lso to carrot by potato psyllid seemingly could easily be adapted to determine whether infected carrot psyllids are able to transmit Lso to potato.

## Supporting Information

S1 Table Alignment of several ‘*Candidatus* Liberibacter solanacearum’ (Lso) sequences obtained from carrot and psyllid samples from U.S., Mexico, and Europe. The sequences are compared to those generated from symptomatic carrots during the present study (GenBank Accession Nos. KU588194 and KU588195). Lso in the present study was amplified using CLi.po.F/OI2c (1071-bp fragment) as described in Secor et al. [29].
(DOCX)

S2 Table Alignment of consensus sequences of *‘Candidatus* Liberibacter solanacearum’ (Lso) obtained from symptomatic carrot samples during the present study (GenBank Accession Nos. KU588194 and KU588195) with those of Lso haplotypes A and B. These generated sequences match 100% Lso haplotype B.
(DOCX)
