# Supplementary material for: Assessing the Likelihood of Transmission of Candidatus Liberibacter solanacearum to Carrot by Potato Psyllid, Bactericera cockerelli (Hemiptera: Triozidae)
Source: PLoS One. 2016 Aug 15;11(8):e0161016. doi: 10.1371/journal.pone.0161016 (PMC4985061; doi:10.1371/journal.pone.0161016)
Supplement: S1 Table — The sequences are compared to those generated from symptomatic carrots during the present study (GenBank Accession Nos. KU588194 and KU588195). Lso in the present study was amplified using CLi.po.F/OI2c (1071-bp fragment) as described in Secor et al. [29]. (DOCX) [file pone.0161016.s001.docx]

**Supplement Table 1. Alignment of several ‘*Candidatus* Liberibacter solanacearum’ (Lso) sequences obtained from carrot and psyllid samples from U.S., Mexico, and Europe. The sequences are compared to those generated from symptomatic carrots during the present study (GenBank Accession Nos. KU588194 and KU588195). Lso in the present study was amplified using CLi.po.F/OI2c (1071-bp fragment) as described in Secor et al. [29]**

Canary_Island_carrot_-_HQ45431 --------------------------------------------------

Psyllids_Canary_HQ454316 --------------------------------------------------

Spain_carrot_HQ454302 --------------------------------------------------

Finland_carrot_GU373049 GCGCTTATTTTTAATAGGAGCGGCAGACGGGTGAGTAACGCGTGGGAATC 50

Sweden_Lso__JN863095_ GCGCTTATTTTTAATAGGAGCGGCAGACGGGTGAGTAACGCGTGGGAATC 50

Finland_Lso__GU373049_ GCGCTTATTTTTAATAGGAGCGGCAGACGGGTGAGTAACGCGTGGGAATC 50

Norway_Lso__JN863097_ GCGCTTATTTTTAATAGGAGCGGCAGACGGGTGAGTAACGCGTGGGAATC 50

Mexico_Potato__FJ498806_ GCGCTTATTTTTAATAGGAGCGGCAGACGGGTGAGTAACGCGTGGGAATC 50

This study__KU588194_ --------------------------------------------------

This study__KU588195_ --------------------------------------------------

Texas_Lso_2013__JX559780_ GCGCTTATTTTTAATAGGAGCGGCAGACGGGTGAGTAACGCGTGGGAATC 50

Washington_Lso__HM245242_ GCGCTTATTTTTAATAGGAGCGGCAGACGGGTGAGTAACGCGTGGGAATC 50

Canary_Island_carrot_-_HQ45431 --------------------CGCATGGAAACGTGTGCTAATACCGTATAC 30

Psyllids_Canary_HQ454316 --------------------CGCATGGAAACGTGTGCTAATACCGTATAC 30

Spain_carrot_HQ454302 --------------------CGCATGGAAACGTGTGCTAATACCGTATAC 30

Finland_carrot_GU373049 TACCTTTTTCTACGGGATAACGCACGGAAACGTGTGCTAATACCGTATAC 100

Sweden_Lso__JN863095_ TACCTTTTTCTACGGGATAACGCACGGAAACGTGTGCTAATACCGTATAC 100

Finland_Lso__GU373049_ TACCTTTTTCTACGGGATAACGCACGGAAACGTGTGCTAATACCGTATAC 100

Norway_Lso__JN863097_ TACCTTTTTCTACGGGATAACGCACGGAAACGTGTGCTAATACCGTATAC 100

Mexico_Potato__FJ498806_ TACCTTTTTCTACGGGATAACGCACGGAAACGTGTGCTAATACCGTATAC 100

This study__KU588194_ -----------------------------------------------TAC 3

This study__KU588195_ -----------------------------------------------TAC 3

Texas_Lso_2013__JX559780_ TACCTTTTTCTACGGGATAACGCACGGAAACGTGTGCTAATACCGTATAC 100

Washington_Lso__HM245242_ TACCTTTTTCTACGGGATAACGCACGGAAACGTGTGCTAATACCGTATAC 100

***

Canary_Island_carrot_-_HQ45431 GCCCTGAGAAGGGGAAAGATTTATTGGAGAGAGATGAGCCCGCGTTAGAT 80

Psyllids_Canary_HQ454316 GCCCTGAGAAGGGGAAAGATTTATTGGAGAGAGATGAGCCCGCGTTAGAT 80

Spain_carrot_HQ454302 GCCCTGAGAAGGGGAAAGATTTATTGGAGAGAGATGAGCCCGCGTTAGAT 80

Finland_carrot_GU373049 GCCCTGAGAAGGGGAAAGATTTATTGGAGAGAGATGAGCCCGCGTTAGAT 150

Sweden_Lso__JN863095_ GCCCTGAGAAGGGGAAAGATTTATTGGAGAGAGATGAGCCCGCGTTAGAT 150

Finland_Lso__GU373049_ GCCCTGAGAAGGGGAAAGATTTATTGGAGAGAGATGAGCCCGCGTTAGAT 150

Norway_Lso__JN863097_ GCCCTGAGAAGGGGAAAGATTTATTGGAGAGAGATGAGCCCGCGTTAGAT 150

Mexico_Potato__FJ498806_ GCCCTGAGAAGGGGAAAGATTTATTGGAGAGAGATGAGCCCGCGTTAGAT 150

This study__KU588194_ GCCCTGAGAAGGGGAAAGATTTATTGGAGAGAGATGAGCCCGCGTTAGAT 53

This study__KU588195_ GCCCTGAGAAGGGGAAAGATTTATTGGAGAGAGATGAGCCCGCGTTAGAT 53

Texas_Lso_2013__JX559780_ GCCCTGAGAAGGGGAAAGATTTATTGGAGAGAGATGAGCCCGCGTTAGAT 150

Washington_Lso__HM245242_ GCCCTGAGAAGGGGAAAGATTTATTGGAGAGAGATGAGCCCGCGTTAGAT 150

**************************************************

Canary_Island_carrot_-_HQ45431 TAGCTAGTTGGTGGGGTAAATGCCTACCAAGGCTACGATCTATAGCTGGT 130

Psyllids_Canary_HQ454316 TAGCTAGTTGGTGGGGTAAATGCCTACCAAGGCTACGATCTATAGCTGGT 130

Spain_carrot_HQ454302 TAGCTAGTTGGTGGGGTAAATGCCTACCAAGGCTACGATCTATAGCTGGT 130

Finland_carrot_GU373049 TAGCTAGTTGGTGGGGTAAATGCCTACCAAGGCTACGATCTATAGCTGGT 200

Sweden_Lso__JN863095_ TAGCTAGTTGGTGGGGTAAATGCCTACCAAGGCTACGATCTATAGCTGGT 200

Finland_Lso__GU373049_ TAGCTAGTTGGTGGGGTAAATGCCTACCAAGGCTACGATCTATAGCTGGT 200

Norway_Lso__JN863097_ TAGCTAGTTGGTGGGGTAAATGCCTACCAAGGCTACGATCTATAGCTGGT 200

Mexico_Potato__FJ498806_ TAGCTAGTTGGTGGGGTAAAGGCCTACCAAGGCTACGATCTATAGCTGGT 200

This study__KU588194_ TAGCTAGTTGGTGGGGTAAAGGCCTACCAAGGCTACGATCTATAGCTGGT 103

This study__KU588195_ TAGCTAGTTGGTGGGGTAAAGGCCTACCAAGGCTACGATCTATAGCTGGT 103

Texas_Lso_2013__JX559780_ TAGCTAGTTGGTGGGGTAAAGGCCTACCAAGGCTACGATCTATAGCTGGT 200

Washington_Lso__HM245242_ TAGCTAGTTGGTGGGGTAAAGGCCTACCAAGGCTACGATCTATAGCTGGT 200

******************** *****************************

Canary_Island_carrot_-_HQ45431 CTGAGAGGACGATCAGCCACACTGGGACTGAGACACGGCCCAGACTCCTA 180

Psyllids_Canary_HQ454316 CTGAGAGGACGATCAGCCACACTGGGACTGAGACACGGCCCAGACTCCTA 180

Spain_carrot_HQ454302 CTGAGAGGACGATCAGCCACACTGGGACTGAGACACGGCCCAGACTCCTA 180

Finland_carrot_GU373049 CTGAGAGGACGATCAGCCACACTGGGACTGAGACACGGCCCAGACTCCTA 250

Sweden_Lso__JN863095_ CTGAGAGGACGATCAGCCACACTGGGACTGAGACACGGCCCAGACTCCTA 250

Finland_Lso__GU373049_ CTGAGAGGACGATCAGCCACACTGGGACTGAGACACGGCCCAGACTCCTA 250

Norway_Lso__JN863097_ CTGAGAGGACGATCAGCCACACTGGGACTGAGACACGGCCCAGACTCCTA 250

Mexico_Potato__FJ498806_ CTGAGAGGACGATCAGCCACACTGGGACTGAGACACGGCCCAGACTCCTA 250

This study__KU588194_ CTGAGAGGACGATCAGCCACACTGGGACTGAGACACGGCCCAGACTCCTA 153

This study__KU588195_ CTGAGAGGACGATCAGCCACACTGGGACTGAGACACGGCCCAGACTCCTA 153

Texas_Lso_2013__JX559780_ CTGAGAGGACGATCAGCCACACTGGGACTGAGACACGGCCCAGACTCCTA 250

Washington_Lso__HM245242_ CTGAGAGGACGATCAGCCACACTGGGACTGAGACACGGCCCAGACTCCTA 250

**************************************************

Canary_Island_carrot_-_HQ45431 CGGGAGGCAGCAGTGGGGAATATTGGACAATGGGGGCAACCCTGATCCAG 230

Psyllids_Canary_HQ454316 CGGGAGGCAGCAGTGGGGAATATTGGACAATGGGGGCAACCCTGATCCAG 230

Spain_carrot_HQ454302 CGGGAGGCAGCAGTGGGGAATATTGGACAATGGGGGCAACCCTGATCCAG 230

Finland_carrot_GU373049 CGGGAGGCAGCAGTGGGGAATATTGGACAATGGGGGCAACCCTGATCCAG 300

Sweden_Lso__JN863095_ CGGGAGGCAGCAGTGGGGAATATTGGACAATGGGGGCAACCCTGATCCAG 300

Finland_Lso__GU373049_ CGGGAGGCAGCAGTGGGGAATATTGGACAATGGGGGCAACCCTGATCCAG 300

Norway_Lso__JN863097_ CGGGAGGCAGCAGTGGGGAATATTGGACAATGGGGGCAACCCTGATCCAG 300

Mexico_Potato__FJ498806_ CGGGAGGCAGCAGTGGGGAATATTGGACAATGGGGGCAACCCTGATCCAG 300

This study__KU588194_ CGGGAGGCAGCAGTGGGGAATATTGGACAATGGGGGCAACCCTGATCCAG 203

This study__KU588195_ CGGGAGGCAGCAGTGGGGAATATTGGACAATGGGGGCAACCCTGATCCAG 203

Texas_Lso_2013__JX559780_ CGGGAGGCAGCAGTGGGGAATATTGGACAATGGGGGCAACCCTGATCCAG 300

Washington_Lso__HM245242_ CGGGAGGCAGCAGTGGGGAATATTGGACAATGGGGGCAACCCTGATCCAG 300

**************************************************

Canary_Island_carrot_-_HQ45431 CCATGCCGCGTGAGTGAAGAAGGCCTTAGGGTTGTAAAGCTCTTTCGCCG 280

Psyllids_Canary_HQ454316 CCATGCCGCGTGAGTGAAGAAGGCCTTAGGGTTGTAAAGCTCTTTCGCCG 280

Spain_carrot_HQ454302 CCATGCCGCGTGAGTGAAGAAGGCCTTAGGGTTGTAAAGCTCTTTCGCCG 280

Finland_carrot_GU373049 CCATGCCGCGTGAGTGAAGAAGGCCTTAGGGTTGTAAAGCTCTTTCGCCG 350

Sweden_Lso__JN863095_ CCATGCCGCGTGAGTGAAGAAGGCCTTAGGGTTGTAAAGCTCTTTCGCCG 350

Finland_Lso__GU373049_ CCATGCCGCGTGAGTGAAGAAGGCCTTAGGGTTGTAAAGCTCTTTCGCCG 350

Norway_Lso__JN863097_ CCATGCCGCGTGAGTGAAGAAGGCCTTAGGGTTGTAAAGCTCTTTCGCCG 350

Mexico_Potato__FJ498806_ CCATGCCGCGTGAGTGAAGAAGGCCTTAGGGTTGTAAAGCTCTTTCGCCG 350

This study__KU588194_ CCATGCCGCGTGAGTGAAGAAGGCCTTAGGGTTGTAAAGCTCTTTCGCCG 253

This study__KU588195_ CCATGCCGCGTGAGTGAAGAAGGCCTTAGGGTTGTAAAGCTCTTTCGCCG 253

Texas_Lso_2013__JX559780_ CCATGCCGCGTGAGTGAAGAAGGCCTTAGGGTTGTAAAGCTCTTTCGCCG 350

Washington_Lso__HM245242_ CCATGCCGCGTGAGTGAAGAAGGCCTTAGGGTTGTAAAGCTCTTTCGCCG 350

**************************************************

Canary_Island_carrot_-_HQ45431 GAGAAGATAATGACGGTATCCGGAGAAGAAGTCCCGGCTAACTTCGTGCC 330

Psyllids_Canary_HQ454316 GAGAAGATAATGACGGTATCCGGAGAAGAAGTCCCGGCTAACTTCGTGCC 330

Spain_carrot_HQ454302 GAGAAGATAATGACGGTATCCGGAGAAGAAGTCCCGGCTAACTTCGTGCC 330

Finland_carrot_GU373049 GAGAAGATAATGACGGTATCCGGAGAAGAAGTCCCGGCTAACTTCGTGCC 400

Sweden_Lso__JN863095_ GAGAAGATAATGACGGTATCCGGAGAAGAAGTCCCGGCTAACTTCGTGCC 400

Finland_Lso__GU373049_ GAGAAGATAATGACGGTATCCGGAGAAGAAGTCCCGGCTAACTTCGTGCC 400

Norway_Lso__JN863097_ GAGAAGATAATGACGGTATCCGGAGAAGAAGTCCCGGCTAACTTCGTGCC 400

Mexico_Potato__FJ498806_ GAGAAGATAATGACGGTATCCGGAGAAGAAGTCCCGGCTAACTTCGTGCC 400

This study__KU588194_ GAGAAGATAATGACGGTATCCGGAGAAGAAGTCCCGGCTAACTTCGTGCC 303

This study__KU588195_ GAGAAGATAATGACGGTATCCGGAGAAGAAGTCCCGGCTAACTTCGTGCC 303

Texas_Lso_2013__JX559780_ GAGAAGATAATGACGGTATCCGGAGAAGAAGTCCCGGCTAACTTCGTGCC 400

Washington_Lso__HM245242_ GAGAAGATAATGACGGTATCCGGAGAAGAAGTCCCGGCTAACTTCGTGCC 400

**************************************************

Canary_Island_carrot_-_HQ45431 AGCAGCCGCGGTAATACGAAGGGGGCGAGCGTTGTTCGGAATAACTGGGC 380

Psyllids_Canary_HQ454316 AGCAGCCGCGGTAATACGAAGGGGGCGAGCGTTGTTCGGAATAACTGGGC 380

Spain_carrot_HQ454302 AGCAGCCGCGGTAATACGAAGGGGGCGAGCGTTGTTCGGAATAACTGGGC 380

Finland_carrot_GU373049 AGCAGCCGCGGTAATACGAAGGGGGCGAGCGTTGTTCGGAATAACTGGGC 450

Sweden_Lso__JN863095_ AGCAGCCGCGGTAATACGAAGGGGGCGAGCGTTGTTCGGAATAACTGGGC 450

Finland_Lso__GU373049_ AGCAGCCGCGGTAATACGAAGGGGGCGAGCGTTGTTCGGAATAACTGGGC 450

Norway_Lso__JN863097_ AGCAGCCGCGGTAATACGAAGGGGGCGAGCGTTGTTCGGAATAACTGGGC 450

Mexico_Potato__FJ498806_ AGCAGCCGCGGTAATACGAAGGGGGCGAGCGTTGTTCGGAATAACTGGGC 450

This study__KU588194_ AGCAGCCGCGGTAATACGAAGGGGGCGAGCGTTGTTCGGAATAACTGGGC 353

This study__KU588195_ AGCAGCCGCGGTAATACGAAGGGGGCGAGCGTTGTTCGGAATAACTGGGC 353

Texas_Lso_2013__JX559780_ AGCAGCCGCGGTAATACGAAGGGGGCGAGCGTTGTTCGGAATAACTGGGC 450

Washington_Lso__HM245242_ AGCAGCCGCGGTAATACGAAGGGGGCGAGCGTTGTTCGGAATAACTGGGC 450

**************************************************

Canary_Island_carrot_-_HQ45431 GTAAAGGGCGCGTAGGCGGGTAATTAAGTTAGGGGTGAAATCCCAAGGCT 430

Psyllids_Canary_HQ454316 GTAAAGGGCGCGTAGGCGGGTAATTAAGTTAGGGGTGAAATCCCAAGGCT 430

Spain_carrot_HQ454302 GTAAAGGGCGCGTAGGCGGGTAATTAAGTTAGGGGTGAAATCCCAAGGCT 430

Finland_carrot_GU373049 GTAAAGGGCGCGTAGGCGGGTAATTAAGTTAGGGGTGAAATCCCAAGGCT 500

Sweden_Lso__JN863095_ GTAAAGGGCGCGTAGGCGGGTAATTAAGTTAGGGGTGAAATCCCAAGGCT 500

Finland_Lso__GU373049_ GTAAAGGGCGCGTAGGCGGGTAATTAAGTTAGGGGTGAAATCCCAAGGCT 500

Norway_Lso__JN863097_ GTAAAGGGCGCGTAGGCGGGTAATTAAGTTAGGGGTGAAATCCCAAGGCT 500

Mexico_Potato__FJ498806_ GTAAAGGGCGCGTAGGCGGGTAATTAAGTTAGGGGTGAAATCCCAAGGCT 500

This study__KU588194_ GTAAAGGGCGCGTAGGCGGGTAATTAAGTTAGGGGTGAAATCCCAAGGCT 403

This study__KU588195_ GTAAAGGGCGCGTAGGCGGGTAATTAAGTTAGGGGTGAAATCCCAAGGCT 403

Texas_Lso_2013__JX559780_ GTAAAGGGCGCGTAGGCGGGTAATTAAGTTAGGGGTGAAATCCCAAGGCT 500

Washington_Lso__HM245242_ GTAAAGGGCGCGTAGGCGGGTAATTAAGTTAGGGGTGAAATCCCAAGGCT 500

**************************************************

Canary_Island_carrot_-_HQ45431 CAACCTTGGAACTGCCTTTAATACTGGTTATCTAGAGTTTAGGAGAGGTG 480

Psyllids_Canary_HQ454316 CAACCTTGGAACTGCCTTTAATACTGGTTATCTAGAGTTTAGGAGAGGTG 480

Spain_carrot_HQ454302 CAACCTTGGAACTGCCTTTAATACTGGTTATCTAGAGTTTAGGAGAGGTG 480

Finland_carrot_GU373049 CAACCTTGGAACTGCCTTTAATACTGGTTATCTAGAGTTTAGGAGAGGTG 550

Sweden_Lso__JN863095_ CAACCTTGGAACTGCCTTTAATACTGGTTATCTAGAGTTTAGGAGAGGTG 550

Finland_Lso__GU373049_ CAACCTTGGAACTGCCTTTAATACTGGTTATCTAGAGTTTAGGAGAGGTG 550

Norway_Lso__JN863097_ CAACCTTGGAACTGCCTTTAATACTGGTTATCTAGAGTTTAGGAGAGGTG 550

Mexico_Potato__FJ498806_ CAACCTTGGAACTGCCTTTAATACTGGTTATCTAGAGTTCAGGAGAGGTG 550

This study__KU588194_ CAACCTTGGAACTGCCTTTAATACTGGTTATCTAGAGTTCAGGAGAGGTG 453

This study__KU588195_ CAACCTTGGAACTGCCTTTAATACTGGTTATCTAGAGTTCAGGAGAGGTG 453

Texas_Lso_2013__JX559780_ CAACCTTGGAACTGCCTTTAATACTGGTTATCTAGAGTTCAGGAGAGGTG 550

Washington_Lso__HM245242_ CAACCTTGGAACTGCCTTTAATACTGGTTATCTAGAGTTCAGGAGAGGTG 550

*************************************** **********

Canary_Island_carrot_-_HQ45431 AGTGGAATTCCGAGTGTAGAGGTGAAATTCGCAGATATTCGGAGGAACAC 530

Psyllids_Canary_HQ454316 AGTGGAATTCCGAGTGTAGAGGTGAAATTCGCAGATATTCGGAGGAACAC 530

Spain_carrot_HQ454302 AGTGGAATTCCGAGTGTAGAGGTGAAATTCGCAGATATTCGGAGGAACAC 530

Finland_carrot_GU373049 AGTGGAATTCCGAGTGTAGAGGTGAAATTCGCAGATATTCGGAGGAACAC 600

Sweden_Lso__JN863095_ AGTGGAATTCCGAGTGTAGAGGTGAAATTCGCAGATATTCGGAGGAACAC 600

Finland_Lso__GU373049_ AGTGGAATTCCGAGTGTAGAGGTGAAATTCGCAGATATTCGGAGGAACAC 600

Norway_Lso__JN863097_ AGTGGAATTCCGAGTGTAGAGGTGAAATTCGCAGATATTCGGAGGAACAC 600

Mexico_Potato__FJ498806_ AGTGGAATTCCGAGTGTAGAGGTGAAATTCGCAGATATTCGGAGGAACAC 600

This study__KU588194_ AGTGGAATTCCGAGTGTAGAGGTGAAATTCGCAGATATTCGGAGGAACAC 503

This study__KU588195_ AGTGGAATTCCGAGTGTAGAGGTGAAATTCGCAGATATTCGGAGGAACAC 503

Texas_Lso_2013__JX559780_ AGTGGAATTCCGAGTGTAGAGGTGAAATTCGCAGATATTCGGAGGAACAC 600

Washington_Lso__HM245242_ AGTGGAATTCCGAGTGTAGAGGTGAAATTCGCAGATATTCGGAGGAACAC 600

**************************************************

Canary_Island_carrot_-_HQ45431 CAGTGGCGAAGGCGGCTCACTGGCCTGATACTGACGCTGAGGCGCGAAAG 580

Psyllids_Canary_HQ454316 CAGTGGCGAAGGCGGCTCACTGGCCTGATACTGACGCTGAGGCGCGAAAG 580

Spain_carrot_HQ454302 CAGTGGCGAAGGCGGCTCACTGGCCTGATACTGACGCTGAGGCGCGAAAG 580

Finland_carrot_GU373049 CAGTGGCGAAGGCGGCTCACTGGCCTGATACTGACGCTGAGGCGCGAAAG 650

Sweden_Lso__JN863095_ CAGTGGCGAAGGCGGCTCACTGGCCTGATACTGACGCTGAGGCGCGAAAG 650

Finland_Lso__GU373049_ CAGTGGCGAAGGCGGCTCACTGGCCTGATACTGACGCTGAGGCGCGAAAG 650

Norway_Lso__JN863097_ CAGTGGCGAAGGCGGCTCACTGGCCTGATACTGACGCTGAGGCGCGAAAG 650

Mexico_Potato__FJ498806_ CAGTGGCGAAGGCGGCTCACTGGCCTGATACTGACGCTGAGGCGCGAAAG 650

This study__KU588194_ CAGTGGCGAAGGCGGCTCACTGGCCTGATACTGACGCTGAGGCGCGAAAG 553

This study__KU588195_ CAGTGGCGAAGGCGGCTCACTGGCCTGATACTGACGCTGAGGCGCGAAAG 553

Texas_Lso_2013__JX559780_ CAGTGGCGAAGGCGGCTCACTGGCCTGATACTGACGCTGAGGCGCGAAAG 650

Washington_Lso__HM245242_ CAGTGGCGAAGGCGGCTCACTGGCCTGATACTGACGCTGAGGCGCGAAAG 650

**************************************************

Canary_Island_carrot_-_HQ45431 CGTGGGGAGCAAACAGGATTAGATACCCTGGTAGTCCACGCTGTAAACGA 630

Psyllids_Canary_HQ454316 CGTGGGGAGCAAACAGGATTAGATACCCTGGTAGTCCACGCTGTAAACGA 630

Spain_carrot_HQ454302 CGTGGGGAGCAAACAGGATTAGATACCCTGGTAGTCCACGCTGTAAACGA 630

Finland_carrot_GU373049 CGTGGGGAGCAAACAGGATTAGATACCCTGGTAGTCCACGCTGTAAACGA 700

Sweden_Lso__JN863095_ CGTGGGGAGCAAACAGGATTAGATACCCTGGTAGTCCACGCTGTAAACGA 700

Finland_Lso__GU373049_ CGTGGGGAGCAAACAGGATTAGATACCCTGGTAGTCCACGCTGTAAACGA 700

Norway_Lso__JN863097_ CGTGGGGAGCAAACAGGATTAGATACCCTGGTAGTCCACGCTGTAAACGA 700

Mexico_Potato__FJ498806_ CGTGGGGAGCAAACAGGATTAGATACCCTGGTAGTCCACGCTGTAAACGA 700

This study__KU588194_ CGTGGGGAGCAAACAGGATTAGATACCCTGGTAGTCCACGCTGTAAACGA 603

This study__KU588195_ CGTGGGGAGCAAACAGGATTAGATACCCTGGTAGTCCACGCTGTAAACGA 603

Texas_Lso_2013__JX559780_ CGTGGGGAGCAAACAGGATTAGATACCCTGGTAGTCCACGCTGTAAACGA 700

Washington_Lso__HM245242_ CGTGGGGAGCAAACAGGATTAGATACCCTGGTAGTCCACGCTGTAAACGA 700

**************************************************

Canary_Island_carrot_-_HQ45431 TGAGTGCTAGCTGTTGGGTGGTTTACCATTCAGTGGCGCAGCTAACGCAT 680

Psyllids_Canary_HQ454316 TGAGTGCTAGCTGTTGGGTGGTTTACCATTCAGTGGCGCAGCTAACGCAT 680

Spain_carrot_HQ454302 TGAGTGCTAGCTGTTGGGTGGTTTACCATTCAGTGGCGCAGCTAACGCAT 680

Finland_carrot_GU373049 TGAGTGCTAGCTGTTGGGTGGTTTACCATTCAGTGGCGCAGCTAACGCAT 750

Sweden_Lso__JN863095_ TGAGTGCTAGCTGTTGGGTGGTTTACCATTCAGTGGCGCAGCTAACGCAT 750

Finland_Lso__GU373049_ TGAGTGCTAGCTGTTGGGTGGTTTACCATTCAGTGGCGCAGCTAACGCAT 750

Norway_Lso__JN863097_ TGAGTGCTAGCTGTTGGGTGGTTTACCATTCAGTGGCGCAGCTAACGCAT 750

Mexico_Potato__FJ498806_ TGAGTGCTAGCTGTTGGGTGGTTTACCATTCAGTGGCGCAGCTAACGCAT 750

This study__KU588194_ TGAGTGCTAGCTGTTGGGTGGTTTACCATTCAGTGGCGCAGCTAACGCAT 653

This study__KU588195_ TGAGTGCTAGCTGTTGGGTGGTTTACCATTCAGTGGCGCAGCTAACGCAT 653

Texas_Lso_2013__JX559780_ TGAGTGCTAGCTGTTGGGTGGTTTACCATTCAGTGGCGCAGCTAACGCAT 750

Washington_Lso__HM245242_ TGAGTGCTAGCTGTTGGGTGGTTTACCATTCAGTGGCGCAGCTAACGCAT 750

**************************************************

Canary_Island_carrot_-_HQ45431 TAAGCACTCCGCCTGGGGAGTACGGTCGCAAGATTAAAACTCAAAGGAAT 730

Psyllids_Canary_HQ454316 TAAGCACTCCGCCTGGGGAGTACGGTCGCAAGATTAAAACTCAAAGGAAT 730

Spain_carrot_HQ454302 TAAGCACTCCGCCTGGGGAGTACGGTCGCAAGATTAAAACTCAAAGGAAT 730

Finland_carrot_GU373049 TAAGCACTCCGCCTGGGGAGTACGGTCGCAAGATTAAAACTCAAAGGAAT 800

Sweden_Lso__JN863095_ TAAGCACTCCGCCTGGGGAGTACGGTCGCAAGATTAAAACTCAAAGGAAT 800

Finland_Lso__GU373049_ TAAGCACTCCGCCTGGGGAGTACGGTCGCAAGATTAAAACTCAAAGGAAT 800

Norway_Lso__JN863097_ TAAGCACTCCGCCTGGGGAGTACGGTCGCAAGATTAAAACTCAAAGGAAT 800

Mexico_Potato__FJ498806_ TAAGCACTCCGCCTGGGGAGTACGGTCGCAAGATTAAAACTCAAAGGAAT 800

This study__KU588194_ TAAGCACTCCGCCTGGGGAGTACGGTCGCAAGATTAAAACTCAAAGGAAT 703

This study__KU588195_ TAAGCACTCCGCCTGGGGAGTACGGTCGCAAGATTAAAACTCAAAGGAAT 703

Texas_Lso_2013__JX559780_ TAAGCACTCCGCCTGGGGAGTACGGTCGCAAGATTAAAACTCAAAGGAAT 800

Washington_Lso__HM245242_ TAAGCACTCCGCCTGGGGAGTACGGTCGCAAGATTAAAACTCAAAGGAAT 800

**************************************************

Canary_Island_carrot_-_HQ45431 TGACGGGGGCCCGCACAAGCGGTGGAGCATGTGGTTTAATTCGATGCAAC 780

Psyllids_Canary_HQ454316 TGACGGGGGCCCGCACAAGCGGTGGAGCATGTGGTTTAATTCGATGCAAC 780

Spain_carrot_HQ454302 TGACGGGGGCCCGCACAAGCGGTGGAGCATGTGGTTTAATTCGATGCAAC 780

Finland_carrot_GU373049 TGACGGGGGCCCGCACAAGCGGTGGAGCATGTGGTTTAATTCGATGCAAC 850

Sweden_Lso__JN863095_ TGACGGGGGCCCGCACAAGCGGTGGAGCATGTGGTTTAATTCGATGCAAC 850

Finland_Lso__GU373049_ TGACGGGGGCCCGCACAAGCGGTGGAGCATGTGGTTTAATTCGATGCAAC 850

Norway_Lso__JN863097_ TGACGGGGGCCCGCACAAGCGGTGGAGCATGTGGTTTAATTCGATGCAAC 850

Mexico_Potato__FJ498806_ TGACGGGGGCCCGCACAAGCGGTGGAGCATGTGGTTTAATTCGATGCAAC 850

This study__KU588194_ TGACGGGGGCCCGCACAAGCGGTGGAGCATGTGGTTTAATTCGATGCAAC 753

This study__KU588195_ TGACGGGGGCCCGCACAAGCGGTGGAGCATGTGGTTTAATTCGATGCAAC 753

Texas_Lso_2013__JX559780_ TGACGGGGGCCCGCACAAGCGGTGGAGCATGTGGTTTAATTCGATGCAAC 850

Washington_Lso__HM245242_ TGACGGGGGCCCGCACAAGCGGTGGAGCATGTGGTTTAATTCGATGCAAC 850

**************************************************

Canary_Island_carrot_-_HQ45431 GCGCAGAACCTTACCAGCCCTTGACATATAGAGGACGATATCAGAGATGG 830

Psyllids_Canary_HQ454316 GCGCAGAACCTTACCAGCCCTTGACATATAGAGGACGATATCAGAGATGG 830

Spain_carrot_HQ454302 GCGCAGAACCTTACCAGCCCTTGACATATAGAGGACGATATCAGAGATGG 830

Finland_carrot_GU373049 GCGCAGAACCTTACCAGCCCTTGACATATAGAGGACGATATCAGAGATGG 900

Sweden_Lso__JN863095_ GCGCAGAACCTTACCAGCCCTTGACATATAGAGGACGATATCAGAGATGG 900

Finland_Lso__GU373049_ GCGCAGAACCTTACCAGCCCTTGACATATAGAGGACGATATCAGAGATGG 900

Norway_Lso__JN863097_ GCGCAGAACCTTACCAGCCCTTGACATATAGAGGACGATATCAGAGATGG 900

Mexico_Potato__FJ498806_ GCGCAGAACCTTACCAGCCCTTGACATATAGAGGACGATATCAGAGATGG 900

This study__KU588194_ GCGCAGAACCTTACCAGCCCTTGACATATAGAGGACGATATCAGAGATGG 803

This study__KU588195_ GCGCAGAACCTTACCAGCCCTTGACATATAGAGGACGATATCAGAGATGG 803

Texas_Lso_2013__JX559780_ GCGCAGAACCTTACCAGCCCTTGACATATAGAGGACGATATCAGAGATGG 900

Washington_Lso__HM245242_ GCGCAGAACCTTACCAGCCCTTGACATATAGAGGACGATATCAGAGATGG 900

**************************************************

Canary_Island_carrot_-_HQ45431 TATTTTCTTTTCGGAGACCTTTATACAGGTGCTGCATGGCTGTCGTCAGC 880

Psyllids_Canary_HQ454316 TATTTTCTTTTCGGAGACCTTTATACAGGTGCTGCATGGCTGTCGTCAGC 880

Spain_carrot_HQ454302 TATTTTCTTTTCGGAGACCTTTATACAGGTGCTGCATGGCTGTCGTCAGC 880

Finland_carrot_GU373049 TATTTTCTTTTCGGAGACCTTTATACAGGTGCTGCATGGCTGTCGTCAGC 950

Sweden_Lso__JN863095_ TATTTTCTTTTCGGAGACCTTTATACAGGTGCTGCATGGCTGTCGTCAGC 950

Finland_Lso__GU373049_ TATTTTCTTTTCGGAGACCTTTATACAGGTGCTGCATGGCTGTCGTCAGC 950

Norway_Lso__JN863097_ TATTTTCTTTTCGGAGACCTTTATACAGGTGCTGCATGGCTGTCGTCAGC 950

Mexico_Potato__FJ498806_ TATTTTCTTTTCGGAGACCTTTATACAGGTGCTGCATGGCTGTCGTCAGC 950

This study__KU588194_ TATTTTCTTTTCGGAGACCTTTATACAGGTGCTGCATGGCTGTCGTCAGC 853

This study__KU588195_ TATTTTCTTTTCGGAGACCTTTATACAGGTGCTGCATGGCTGTCGTCAGC 853

Texas_Lso_2013__JX559780_ TATTTTCTTTTCGGAGACCTTTATACAGGTGCTGCATGGCTGTCGTCAGC 950

Washington_Lso__HM245242_ TATTTTCTTTTCGGAGACCTTTATACAGGTGCTGCATGGCTGTCGTCAGC 950

**************************************************

Canary_Island_carrot_-_HQ45431 TCGTGTCGTGAGATGTTGGGTTAAGTCCCGCAACGAGCGCAACCCCTGCC 930

Psyllids_Canary_HQ454316 TCGTGTCGTGAGATGTTGGGTTAAGTCCCGCAACGAGCGCAACCCCTGCC 930

Spain_carrot_HQ454302 TCGTGTCGTGAGATGTTGGGTTAAGTCCCGCAACGAGCGCAACCCCTGCC 930

Finland_carrot_GU373049 TCGTGTCGTGAGATGTTGGGTTAAGTCCCGCAACGAGCGCAACCCCTGCC 1000

Sweden_Lso__JN863095_ TCGTGTCGTGAGATGTTGGGTTAAGTCCCGCAACGAGCGCAACCCCTGCC 1000

Finland_Lso__GU373049_ TCGTGTCGTGAGATGTTGGGTTAAGTCCCGCAACGAGCGCAACCCCTGCC 1000

Norway_Lso__JN863097_ TCGTGTCGTGAGATGTTGGGTTAAGTCCCGCAACGAGCGCAACCCCTGCC 1000

Mexico_Potato__FJ498806_ TCGTGTCGTGAGATGTTGGGTTAAGTCCCGCAACGAGCGCAACCCCTACC 1000

This study__KU588194_ TCGTGTCGTGAGATGTTGGGTTAAGTCCCGCAACGAGCGCAACCCCTACC 903

This study__KU588195_ TCGTGTCGTGAGATGTTGGGTTAAGTCCCGCAACGAGCGCAACCCCTACC 903

Texas_Lso_2013__JX559780_ TCGTGTCGTGAGATGTTGGGTTAAGTCCCGCAACGAGCGCAACCCCTACC 1000

Washington_Lso__HM245242_ TCGTGTCGTGAGATGTTGGGTTAAGTCCCGCAACGAGCGCAACCCCTACC 1000

*********************************************** **

Canary_Island_carrot_-_HQ45431 TCTAGTTGCCATCAAGTTTAGATTTTATCTAAATGTTGGGTACTTTATAG 980

Psyllids_Canary_HQ454316 TCTAGTTGCCATCAAGTTTAGATTTTATCTAAATGTTGGGTACTTTATAG 980

Spain_carrot_HQ454302 TCTAGTTGCCATCAAGTTTAGATTTTATCTAAATGTTGGGTACTTTATAG 980

Finland_carrot_GU373049 TCTAGTTGCCATCAAGTTTAGATTTTATCTAGATGTTGGGTACTTTATAG 1050

Sweden_Lso__JN863095_ TCTAGTTGCCATCAAGTTTAGATTTTATCTAGATGTTGGGTACTTTATAG 1050

Finland_Lso__GU373049_ TCTAGTTGCCATCAAGTTTAGATTTTATCTAGATGTTGGGTACTTTATAG 1050

Norway_Lso__JN863097_ TCTAGTTGCCATCAAGTTTAGATTTTATCTAGATGTTGGGTACTTTATAG 1050

Mexico_Potato__FJ498806_ TCTAGTTGCCATCAAGTTTAGATTTTATCTAGATGTTGGGTACTTTATAG 1050

This study__KU588194_ TCTAGTTGCCATCAAGTTTAGATTTTATCTAGATGTTGGGTACTTTATAG 953

This study__KU588195_ TCTAGTTGCCATCAAGTTTAGATTTTATCTAGATGTTGGGTACTTTATAG 953

Texas_Lso_2013__JX559780_ TCTAGTTGCCATCAAGTTTAGATTTTATCTAGATGTTGGGTACTTTATAG 1050

Washington_Lso__HM245242_ TCTAGTTGCCATCAAGTTTAGATTTTATCTAGATGTTGGGTACTTTATAG 1050

******************************* ******************

Canary_Island_carrot_-_HQ45431 GGACTGCCGGTGATAATCCGGAGGAAGGTGGGGATGACGTC--------- 1021

Psyllids_Canary_HQ454316 GGACTGCCGGTGATAATCCGGAGGAAGGTGGGGATGACGTC--------- 1021

Spain_carrot_HQ454302 GGACTGCCGGTGATAATCCGGAGGAAGGTGGGGATGACGTC--------- 1021

Finland_carrot_GU373049 GGACTGCCGGTGATAATCCGGAGGAAGGTGGGGATGACGTCAAGTCCTCA 1100

Sweden_Lso__JN863095_ GGACTGCCGGTGATAATCCGGAGGAAGGTGGGGATGACGTCAAGTCCTCA 1100

Finland_Lso__GU373049_ GGACTGCCGGTGATAATCCGGAGGAAGGTGGGGATGACGTCAAGTCCTCA 1100

Norway_Lso__JN863097_ GGACTGCCGGTGATAATCCGGAGGAAGGTGGGGATGACGTCAAGTCCTCA 1100

Mexico_Potato__FJ498806_ GGACTGCCGGTGATAATCCGGAGGAAGGTGGGGATGACGTCAAGTCCTCA 1100

This study__KU588194_ GGACTGCCGGTGATAATCCGGAGGAAGGTGGGGATGACGTCAAGTCCTCA 1003

This study__KU588195_ GGACTGCCGGTGATAATCCGGAGGAAGGTGGGGATGACGTCAAGTCCTCA 1003

Texas_Lso_2013__JX559780_ GGACTGCCGGTGATAATCCGGAGGAAGGTGGGGATGACGTCAAGTCCTCA 1100

Washington_Lso__HM245242_ GGACTGCCGGTGATAATCCGGAGGAAGGTGGGGATGACGTCAAGTCCTCA 1100

*****************************************

Canary_Island_carrot_-_HQ45431 --------------------------------------------------

Psyllids_Canary_HQ454316 --------------------------------------------------

Spain_carrot_HQ454302 --------------------------------------------------

Finland_carrot_GU373049 TGGCCCTTATGGGCTGGGCTACACACGTGCTACAATGGTGGTTACAATGG 1150

Sweden_Lso__JN863095_ TGGCCCTTATGGGCTGGGCTACACACGTGCTACAATGGTGGTTACAATGG 1150

Finland_Lso__GU373049_ TGGCCCTTATGGGCTGGGCTACACACGTGCTACAATGGTGGTTACAATGG 1150

Norway_Lso__JN863097_ TGGCCCTTATGGGCTGGGCTACACACGTGCTACAATGGTGGTTACAATGG 1150

Mexico_Potato__FJ498806_ TGGCCCTTATGGGCTGGGCTACACACGTGCTACAATGGTGGTTACAATGG 1150

This study__KU588194_ TGGCCCTTATGGGCTGGGCTACACACGTGCTACAATGGTGGTTACAATGG 1053

This study__KU588195_ TGGCCCTTATGGGCTGGGCTACACACGTGCTACAATGGTGGTTACAATGG 1053

Texas_Lso_2013__JX559780_ TGGCCCTTATGGGCTGGGCTACACACGTGCTACAATGGTGGTTACAATGG 1150

Washington_Lso__HM245242_ TGGCCCTTATGGGCTGGGCTACACACGTGCTACAATGGTGGTTACAATGG 1150

Canary_Island_carrot_-_HQ45431 ------------------

Psyllids_Canary_HQ454316 ------------------

Spain_carrot_HQ454302 ------------------

Finland_carrot_GU373049 GTTGCGAAGTCGCGAGGC 1168

Sweden_Lso__JN863095_ GTTGCGAAGTCGCGAGGC 1168

Finland_Lso__GU373049_ GTTGCGAAGTCGCGAGGC 1168

Norway_Lso__JN863097_ GTTGCGAAGTCGCGAGGC 1168

Mexico_Potato__FJ498806_ GTTGCGAAGTCGCGAGGC 1168

This study__KU588194_ GTTGCGAAGTCGCGAGGC 1071

This study__KU588195_ GTTGCGAAGTCGCGAGGC 1071

Texas_Lso_2013__JX559780_ GTTGCGAAGTCGCGAGGC 1168

Washington_Lso__HM245242_ GTTGCGAAGTCGCGAGGC 1168
