# Supplementary material for: Assessing the Likelihood of Transmission of Candidatus Liberibacter solanacearum to Carrot by Potato Psyllid, Bactericera cockerelli (Hemiptera: Triozidae)
Source: PLoS One. 2016 Aug 15;11(8):e0161016. doi: 10.1371/journal.pone.0161016 (PMC4985061; doi:10.1371/journal.pone.0161016)
Supplement: S2 Table — Alignment of consensus sequences of ‘Candidatus Liberibacter solanacearum’ (Lso) obtained from symptomatic carrot samples during the present study (GenBank Accession Nos. KU588194 and KU588195) with those of Lso haplotypes A and B. These generated sequences match 100% Lso haplotype B. (DOCX) [file pone.0161016.s002.docx]

**Supplement Table 2. Alignment of consensus sequences of ‘*Candidatus* Liberibacter solanacearum’ (Lso) obtained from symptomatic carrot samples during the present study (GenBank Accession Nos. KU588194 and KU588195) with those of Lso haplotypes A and B. These generated sequences match 100% Lso haplotype B.**

LsoA_16S cagaacgaacgctggcggcaggcttaacacatgcaagtcgagcgcttatttttaatagga

LsoB_16S --------------------------------------cgagcgcttatttttaatagga

KU588194 ------------------------------------------------------------

KU588195 -----------------------------------------GCGCTTATTTTTAATAGGA

LsoA_16S gcggcagacgggtgagtaacgcgtgggaatctacctttttctacgggataacgcacggaa

LsoB_16S gcggcagacgggtgagtaacgcgtgggaatctacctttttctacgggataacgcacggaa

KU588194 ------------------------------------------------------------

KU588195 GCGGCAGACGGGTGAGTAACGCGTGGGAATCTACCTTTTTCTACGGGATAACGCACGGAA

LsoA_16S acgtgtgctaataccgtatacgccctgagaaggggaaagatttattggagagagatgagc

LsoB_16S acgtgtgctaataccgtatacgccctgagaaggggaaagatttattggagagagatgagc

KU588194 ------------------TACGCCCTGAGAAGGGGAAAGATTTATTGGAGAGAGATGAGC

KU588195 ACGTGTGCTAATACCGTATACGCCCTGAGAAGGGGAAAGATTTATTGGAGAGAGATGAGC

************************************************************

LsoA_16S ccgcgttagattagctagttggtggggtaaatgcctaccaaggctacgatctatagctgg

LsoB_16S ccgcgttagattagctagttggtggggtaaaggcctaccaaggctacgatctatagctgg

KU588194 CCGCGTTAGATTAGCTAGTTGGTGGGGTAAAGGCCTACCAAGGCTACGATCTATAGCTGG

KU588195 CCGCGTTAGATTAGCTAGTTGGTGGGGTAAAGGCCTACCAAGGCTACGATCTATAGCTGG

******************************* ****************************

LsoA_16S tctgagaggacgatcagccacactgggactgagacacggcccagactcctacgggaggca

LsoB_16S tctgagaggacgatcagccacactgggactgagacacggcccagactcctacgggaggca

KU588194 TCTGAGAGGACGATCAGCCACACTGGGACTGAGACACGGCCCAGACTCCTACGGGAGGCA

KU588195 TCTGAGAGGACGATCAGCCACACTGGGACTGAGACACGGCCCAGACTCCTACGGGAGGCA

************************************************************

LsoA_16S gcagtggggaatattggacaatgggggcaaccctgatccagccatgccgcgtgagtgaag

LsoB_16S gcagtggggaatattggacaatgggggcaaccctgatccagccatgccgcgtgagtgaag

KU588194 GCAGTGGGGAATATTGGACAATGGGGGCAACCCTGATCCAGCCATGCCGCGTGAGTGAAG

KU588195 GCAGTGGGGAATATTGGACAATGGGGGCAACCCTGATCCAGCCATGCCGCGTGAGTGAAG

************************************************************

LsoA_16S aaggccttagggttgtaaagctctttcgccggagaagataatgacggtatccggagaaga

LsoB_16S aaggccttagggttgtaaagctctttcgccggagaagataatgacggtatccggagaaga

KU588194 AAGGCCTTAGGGTTGTAAAGCTCTTTCGCCGGAGAAGATAATGACGGTATCCGGAGAAGA

KU588195 AAGGCCTTAGGGTTGTAAAGCTCTTTCGCCGGAGAAGATAATGACGGTATCCGGAGAAGA

************************************************************

LsoA_16S agtcccggctaacttcgtgccagcagccgcggtaatacgaagggggcgagcgttgttcgg

LsoB_16S agtcccggctaacttcgtgccagcagccgcggtaatacgaagggggcgagcgttgttcgg

KU588194 AGTCCCGGCTAACTTCGTGCCAGCAGCCGCGGTAATACGAAGGGGGCGAGCGTTGTTCGG

KU588195 AGTCCCGGCTAACTTCGTGCCAGCAGCCGCGGTAATACGAAGGGGGCGAGCGTTGTTCGG

************************************************************

LsoA_16S aataactgggcgtaaagggcgcgtaggcgggtaattaagttaggggtgaaatcccaaggc

LsoB_16S aataactgggcgtaaagggcgcgtaggcgggtaattaagttaggggtgaaatcccaaggc

KU588194 AATAACTGGGCGTAAAgGGcGCGTAGGCGGGTAATTAAGTTAGGGGTGAAATCCCAAGGC

KU588195 AATAACTGGGCGTAAAGGGCGCGTAGGCGGGTAATTAAGTTaGGGGTGAAATCCCAAGGC

************************************************************

LsoA_16S tcaaccttggaactgcctttaatactggttatctagagtttaggagaggtgagtggaatt

LsoB_16S tcaaccttggaactgcctttaatactggttatctagagttcaggagaggtgagtggaatt

KU588194 TCAACCTTGGAACTGCCTTTAATACTGGTTATCTAGAGTTCAGGAGAGGTGAGTGGAATT

KU588195 TCAACCTTGGAACTGCCTTTAATACTGGTTATCTAGAGTTCAGGAGAGGTGAGTGGAATT

************************************************************

LsoA_16S ccgagtgtagaggtgaaattcgcagatattcggaggaacaccagtggcgaaggcggctca

LsoB_16S ccgagtgtagaggtgaaattcgcagatattcggaggaacaccagtggcgaaggcggctca

KU588194 CCGAGTGTAGAGGTGAAATTCGCAGATATTCGGAGGAACACCAGTGGCGAAGGCGGCTCA

KU588195 CCGAGTGTAGAGGTGAAATTCGCAGATATTCGGAGGAACACCAGTGGCGAAGGCGGCTCA

************************************************************

LsoA_16S ctggcctgatactgacgctgaggcgcgaaagcgtggggagcaaacaggattagataccct

LsoB_16S ctggcctgatactgacgctgaggcgcgaaagcgtggggagcaaacaggattagataccct

KU588194 CTGGCCTGATACTGACGCTGAGGCGCGAAAGCGTGGGGAGCAAACAGGATTAGATACCCT

KU588195 CTGGCCTGATACTGACGCTGAGGCGCGAAAGCGTGGGGAGCAAACAGGATTAGATACCCT

************************************************************

LsoA_16S ggtagtccacgctgtaaacgatgagtgctagctgttgggtggtttaccattcagtggcgc

LsoB_16S ggtagtccacgctgtaaacgatgagtgctagctgttgggtggtttaccattcagtggcgc

KU588194 GGTAGTCCACGCTGTAAACGATGAGTGCTAGCTGTTGGGTGGTTTACCATTCAGTGGCGC

KU588195 GGTAGTCCACGCTGTAAACGATGAGTGCTAGCTGTTGGGTGGTTTACCATTCAGTGGCGC

************************************************************

LsoA_16S agctaacgcattaagcactccgcctggggagtacggtcgcaagattaaaactcaaaggaa

LsoB_16S agctaacgcattaagcactccgcctggggagtacggtcgcaagattaaaactcaaaggaa

KU588194 AGCTAACGCATTAAGCACTCCGCCTGGGGAGTACGGTCGCAAGATTAAAACTCAAAGGAA

KU588195 AGCTAACGCATTAAGCACTCCGCCTGGGGAGTACGGTCGCAAGATTAAAACTCAAAGGAA

************************************************************

LsoA_16S ttgacgggggcccgcacaagcggtggagcatgtggtttaattcgatgcaacgcgcagaac

LsoB_16S ttgacgggggcccgcacaagcggtggagcatgtggtttaattcgatgcaacgcgcagaac

KU588194 TTGACGGGGGCCCGCACAAGCGGTGGAGCATGTGGTTTAATTCGATGCAACGCGCAGAAC

KU588195 TTGACGGGGGCCCGCACAAGCGGTGGAGCATGTGGTTTAATTCGATGCAACGCGCAGAAC

************************************************************

LsoA_16S cttaccagcccttgacatatagaggacgatatcagagatggtattttcttttcggagacc

LsoB_16S cttaccagcccttgacatatagaggacgatatcagagatggtattttcttttcggagacc

KU588194 CTTACCAGCCCTTGACATATAGAGGACGATATCAGAGATGGTATTTTCTTTTCGGAGACC

KU588195 CTTACCAGCCCTTGACATATAGAGGACGATATCAGAGATGGTATTTTCTTTTCGGAGACC

************************************************************

LsoA_16S tttatacaggtgctgcatggctgtcgtcagctcgtgtcgtgagatgttgggttaagtccc

LsoB_16S tttatacaggtgctgcatggctgtcgtcagctcgtgtcgtgagatgttgggttaagtccc

KU588194 TTTATACAGGTGCTGCATGGCTGTCGTCAGCTCGTGTCGTGAGATGTTGGGTTAAGTCCC

KU588195 TTTATACAGGTGCTGCATGGCTGTCGTCAGCTCGTGTCGTGAGATGTTGGGTTAAGTCCC

************************************************************

LsoA_16S gcaacgagcgcaacccctacctctagttgccatcaagtttagattttatctagatgttgg

LsoB_16S gcaacgagcgcaacccctacctctagttgccatcaagtttagattttatctagatgttgg

KU588194 GCAACGAGCGCAACCCCTACCTCTAGTTGCCATCAAGTTTAGATTTTATCTAGATGTTGG

KU588195 GCAACGAGCGCAACCCCTACCTCTAGTTGCCATCAAGTTTAGATTTTATCTAGATGTTGG

************************************************************

LsoA_16S gtactttatagggactgccggtgataatccggaggaaggtggggatgacgtcaagtcctc

LsoB_16S gtactttatagggactgccggtgataatccggaggaaggtggggatgacgtcaagtcctc

KU588194 GTACTTTATAGGGACTGCCGGTGATAATCCGGAGGAAGGTGGGGATGACGTCAAGTCCTC

KU588195 GTACTTTATAGGGACTGCCGGTGATAATCCGGAGGAAGGTGGGGATGACGTCAAGTCCTC

************************************************************

LsoA_16S atggcccttatgggctgggctacacacgtgctacaatggtggttacaatgggttgcgaag

LsoB_16S atggcccttatgggctgggctacacacgtgctacaatggtggttacaatgggttgcgaag

KU588194 ATGGCCCTTATGGGCTGGGCTACACACGTGCTACAATGGTGGTTACAATGGGTTGCGAAG

KU588195 ATGGCCCTTATGGGCTGGGCTACACACGTGCTACAATGGTGGTTACAATGGGTTGCGAAG

************************************************************

LsoA_16S tcgcgaggcggagctaatcccaaaaggccatctcagttcggattgcactctgcaactcga

LsoB_16S tcgcgaggc---------------------------------------------------

KU588194 TCGCGAGGC---------------------------------------------------

KU588195 TCGCGAGGC---------------------------------------------------

*********
